# Technical note: AQMEII4 Activity 1: evaluation of wet and dry deposition schemes as an integral part of regional-scale air quality models

**DOI:** 10.5194/acp-21-15663-2021

**Published:** 2021-10-20

**Authors:** Stefano Galmarini, Paul Makar, Olivia E. Clifton, Christian Hogrefe, Jesse O. Bash, Roberto Bellasio, Roberto Bianconi, Johannes Bieser, Tim Butler, Jason Ducker, Johannes Flemming, Alma Hodzic, Christopher D. Holmes, Ioannis Kioutsioukis, Richard Kranenburg, Aurelia Lupascu, Juan Luis Perez-Camanyo, Jonathan Pleim, Young-Hee Ryu, Roberto San Jose, Donna Schwede, Sam Silva, Ralf Wolke

**Affiliations:** 1 Joint Research Centre, European Commission, Ispra, Italy; 2 Air Quality Modelling and Integration Section, Environment and Climate Change Canada, Toronto, Canada; 3 National Center for Atmospheric Research, Boulder, CO, USA; 4 Office of Research and Development, U.S. Environmental Protection Agency, Research Triangle Park, NC, USA; 5 Enviroware srl, Concorezzo, MB, Italy; 6 Institute of Coastal Research, Helmholtz-Zentrum Geesthacht, Geesthacht, Germany; 7 Institute for Advanced Sustainability Studies, Potsdam, Germany; 8 Earth, Ocean and Atmospheric Science, Florida State University, Tallahassee, FL, USA; 9 European Centre for Medium-Range Weather Forecasts, Reading, UK; 10 Laboratory of Atmospheric Physics, Department of Physics, University of Patras, Patras, Greece; 11 Netherlands Organization for Applied Scientific Research (TNO), Utrecht, the Netherlands; 12 Technical University of Madrid (UPM), Madrid, Spain; 13 Pohang University of Science and Technology (POSTECH), Pohang, South Korea; 14 Pacific Northwest National Laboratory, Richland, WA, USA; 15 Leibniz Institute for Tropospheric Research, Leipzig, Germany; 16 NASA Goddard Institute for Space Studies, New York, NY, USA

## Abstract

We present in this technical note the research protocol for phase 4 of the Air Quality Model Evaluation International Initiative (AQMEII4). This research initiative is divided into two activities, collectively having three goals: (i) to define the current state of the science with respect to representations of wet and especially dry deposition in regional models, (ii) to quantify the extent to which different dry deposition parameterizations influence retrospective air pollutant concentration and flux predictions, and (iii) to identify, through the use of a common set of detailed diagnostics, sensitivity simulations, model evaluation, and reduction of input uncertainty, the specific causes for the current range of these predictions. Activity 1 is dedicated to the diagnostic evaluation of wet and dry deposition processes in regional air quality models (described in this paper), and Activity 2 to the evaluation of dry deposition point models against ozone flux measurements at multiple towers with multiyear observations (to be described in future submissions as part of the special issue on AQMEII4). The scope of this paper is to present the scientific protocols for Activity 1, as well as to summarize the technical information associated with the different dry deposition approaches used by the participating research groups of AQMEII4. In addition to describing all common aspects and data used for this multi-model evaluation activity, most importantly, we present the strategy devised to allow a common process-level comparison of dry deposition obtained from models using sometimes very different dry deposition schemes. The strategy is based on adding detailed diagnostics to the algorithms used in the dry deposition modules of existing regional air quality models, in particular archiving diagnostics specific to land use–land cover (LULC) and creating standardized LULC categories to facilitate cross-comparison of LULC-specific dry deposition parameters and processes, as well as archiving effective conductance and effective flux as means for comparing the relative influence of different pathways towards the net or total dry deposition. This new approach, along with an analysis of precipitation and wet deposition fields, will provide an unprecedented process-oriented comparison of deposition in regional air quality models. Examples of how specific dry deposition schemes used in participating models have been reduced to the common set of comparable diagnostics defined for AQMEII4 are also presented.

## 1 Introduction

Since 2009, the Air Quality Model Evaluation International Initiative (AQMEII; Rao et al., 2011) has focused on evaluating regional-scale air quality models used for research and regulatory applications. The goal of AQMEII is to conduct coordinated research projects and model inter-comparisons to advance model evaluation practices and inform model development. This initiative is promoted by the European Commission Joint Research Center, the U.S. Environmental Protection Agency (EPA), and Environment and Climate Change Canada and involves the regional-scale air quality research communities active in both North America and Europe.

AQMEII has been executed in phases that each focused on a critical aspect of modelling systems. The phases were conducted as multi-model comparisons that were analysed through the organization of common modelling activities and supported by gathering specific monitoring data needed to evaluate model performance. Each of the phases required developing innovative evaluation and data reconciliation techniques to provide scientific insight across disparate modelling systems. AQMEII phase 1 provided the first detailed annual ensemble comparison of air quality model predictions for North America and Europe (Galmarini et al., 2012). AQMEII phase 2 examined the impacts of feedbacks between air quality and weather on forecasting skill and identified the key sources of uncertainty in feedback model forecasts (Galmarini et al., 2015). AQMEII phase 3, in collaboration with the Task Force on Hemispheric Transport of Air Pollution (TF HTAP) (http://www.htap.org, last access: 7 October 2021), studied the effects of intercontinental transport on regional air quality predictions (Galmarini et al., 2017). Details and findings of the past three phases of AQMEII can be found in journal special issues dedicated to these activities (Galmarini et al., 2012, 2015, 2017). The AQMEII initiative is based on the four pillars of model evaluation described by Dennis et al. (2010): operational, diagnostic, dynamic, and probabilistic evaluation, which will be partly described hereinafter.

This fourth phase of AQMEII (AQMEII4), detailed in this special issue and introduced by a pair of technical notes, focuses on the processes of wet and especially dry deposition, including the parameterized approaches used within current air quality models, and how these approaches and the details of their implementation influence model predictions and performance across multiple modelling systems. Deposition is critical to the life cycle of a pollutant, as it regulates the rate of pollutant removal from the atmosphere and determines the net flux of that pollutant to the earth’s surface. This latter point is particularly important when the pollutants have a known deleterious effect on ecosystems (e.g. the deposition of acidifying compounds to aquatic ecosystems, or the dry deposition of ozone on vegetation). By affecting the pollution remaining in the atmosphere, deposition estimates also modulate predictions of ambient pollutant concentrations that affect human health through inhalation exposure.

Deposition has only been peripherally investigated in past phases of AQMEII. The operational evaluation of air quality models, in which modelled concentrations are directly compared to monitoring network observations, quantifies the extent to which an air quality model meets expected performance. However, operational evaluation does not provide the process-level understanding of the extent to which the performance results from correct representation of model physical and chemical processes. In this context, dry and wet deposition are key processes within air quality models because they represent removal, which can affect the concentrations of key atmospheric species. Several past AQMEII publications were dedicated specifically to wet and dry deposition (Vivanco et al., 2018; Hogrefe et al., 2020; Solazzo et al., 2018). However, only wet deposition fluxes could be evaluated against observational data in these papers. The causes of differences in model predictions for dry deposition were not determined. Some of the studies performed within AQMEII also addressed dynamic evaluation (i.e. the performance of a model in capturing changes in concentrations or deposition fluxes when subjected to variations in meteorology or emissions). The effects of these variations on deposition were therefore investigated, but without analysis at the process level on the extent to which the details of deposition algorithms influenced model performance.

Recent studies of dry deposition of ozone have been fuelled by the need to quantify impacts on global to regional water and carbon cycles (Lombardozzi et al., 2015; Oliver et al., 2018), vegetation damage including crop yields (McGrath et al., 2015; Emberson et al., 2018; Schiferl and Heald, 2018; Hong et al., 2020), and ozone air pollution (Andersson and Engardt, 2010; Silva and Heald, 2018; Baublitz et al., 2020). In particular, reduced stomatal dry deposition of ozone during droughts may contribute to high-ozone-pollution episodes (Vautard et al., 2005; Solberg et al., 2008; Emberson et al., 2013; Huang et al., 2016; Anav et al., 2018; Lin et al., 2020). Dry deposition of ozone occurring through nonstomatal deposition pathways, on average 45 % of the total (Clifton et al., 2020a), has also been shown to be more variable and more important than predicted by current chemical transport models, with implications for background and extreme ozone pollution (Clifton et al., 2017, 2020b). Previous intercomparisons at the global scale suggest large differences in simulated ozone deposition velocities with implications for the simulated tropospheric ozone budgets and the models’ ability to quantitatively capture the drivers of recent trends and interannual variability in observed ozone pollution (Hardacre et al., 2015; Wong et al., 2019). However, process-oriented evaluation in regional to global models is missing, in large part because key process-oriented diagnostics have not been archived and different land use–land cover (LULC) inputs across models have inhibited the systematic elucidation of processes driving the noted differences (Hardacre et al., 2015; Clifton et al., 2020a). One way in which discrepancies between observed and modelled deposition has been addressed is through model–measurement fusion approaches (Schwede and Lear, 2014; Makar et al., 2018; Robichaud et al., 2020a, b). Such approaches could benefit from an improved characterization of process-level uncertainty in modelled dry deposition.

Despite the great advancements in regional-scale air quality modelling, the primary schemes used for dry and wet deposition in today’s m6odels originated in the 1980s and 1990s. Moreover, while the role of deposition as a persistent sink has been known for a long time (e.g. Chang et al., 1987; Irving and Smith, 1991; Borrell and Borrell, 2000), its relative importance in regulating trace species budgets has become more prominent in recent years as the magnitude of the anthropogenic emission source term has generally decreased. The evaluation studies performed within AQMEII (e.g. Solazzo et al., 2017; Hogrefe et al., 2018) and other recent work reaffirmed that deposition is a process of paramount importance within an air quality model (e.g. Knote et al., 2015; Huang et al., 2016; Beddows et al., 2017; Matichuk et al., 2017; Campbell et al., 2019; Sharma et al., 2020) with consequences of primary relevance in a number of sectors (human health, agriculture, forestry, hydrology, soil management, ecosystem management). Thus, there is renewed focus on better characterization of this term and its magnitude.

All the above points were the motivation to make use of the AQMEII community and evaluation infrastructure to construct an AQMEII phase dedicated to deposition. This phase was designed to compare deposition predictions from multiple regional models by isolating specific deposition pathways across multiple modelling systems and across multiple LULC classification systems using common diagnostic tools. Analysing dry deposition of gaseous species, especially ozone and nitrogen species, is a particular focus, as is quantifying the range of model predictions for acidifying wet and dry deposition. A process-level diagnostic intercomparison of particle dry deposition is not conducted here due to the complexity added by model-to-model differences in the representation of aerosols (size and composition) themselves. We also note that some previous work (e.g. Makar et al., 2018) suggests that the impact of particle deposition on total nitrogen and sulfur deposition is relatively small, although particle deposition is the main source of base cations transferred from the atmosphere to ecosystems. However, more recent work (Saylor et al., 2019; Emerson et al., 2020) suggests that particle dry deposition algorithms used in current modelling systems are highly uncertain, suggesting a need for performing further process-level diagnostic intercomparisons.

AQMEII4 has the following research goals.

- Quantify the performance and variability of dry and wet deposition fields simulated by multiple state-of-the-science regional air quality models.
- Document deposition schemes and key parameters used in these models in a framework that allows their easy intercomparison.
- Identify and quantify the causes of differences in modelgenerated deposition fluxes by using detailed ancillary diagnostic fields added to deposition algorithms and common LULC categories.
- Analyse dry deposition module performance with single-point model simulations driven by observation data collected at towers with ozone flux measurements and quantify the impacts of different conditions, processes, and parameters on simulated dry deposition (Activity 2; to be covered in other AQMEII4 special issue publications).
- Investigate methods for using simulated meteorological, concentration, and deposition fields from multiple models in conjunction with available observations to estimate maps of total deposition and their environmental impacts, including the prediction of exceedances of critical loads.

Most model dry deposition schemes are derived from Wesely (1989). However, their implementation in regional and global models has considerable variation (a comparison with global models may be found in Hardacre et al., 2015). Specifically, most schemes follow the parameterization structure used by Wesely (1989) but may differ in the details of their representation of individual parameters and processes. This is discussed in more detail in Sect. 3.

In addition, dry deposition algorithms require, as a key input, information on LULC and vegetation. It is therefore important to determine how the deposition modules themselves work, both as stand-alone physical descriptions and within a regional air quality model. AQMEII4 has been organized as two parallel activities to address the research goals outlined above. AQMEII4 Activity 1 (introduced in this technical note) focuses on the detailed diagnostic comparison of predictions of air quality model deposition fields, along with evaluation of model concentration and wet deposition flux performance at routine monitoring stations in North America (NA) and Europe (EU). Activity 2 (introduced in separate special issue publications) evaluates only the dry deposition schemes used in air quality models, and other models used for impact assessments, such as zero-dimensional single-point models, driven by observed meteorology, biophysics, and ecosystem characteristics, at specific sites across the Northern Hemisphere where ozone flux measurements have been collected continuously over at least a year, with many datasets spanning 3 years or more. AQMEII4 will provide the most comprehensive analyses yet performed on dry deposition schemes, since the schemes will be tested both within and independently from the air quality model, under controlled conditions, and when subjected to variable meteorological and surface characteristic conditions. The single-point modelling component allows a very detailed analysis of how ozone dry deposition is modelled; recent work comparing five deposition algorithms at a single site (Wu et al., 2018) here has been extended to multiple sites with additional deposition algorithms and takes advantage of a new collection of ozone flux measurements at sites around the Northern Hemisphere and new process-oriented diagnostics.

This technical note is designed to summarize all relevant information that constitutes the set-up and organization of AQMEII4 Activity 1. Its intent is to provide both the readers and authors of this special issue with a common reference for the description of the AQMEII4 aims, scientific protocols, and analysis approaches; the model reporting framework; the model input data and monitoring data used for model evaluation; and the descriptions of the model deposition algorithms themselves. By serving as common point of reference for the individual studies undertaken through AQMEII4 Activity 1, the technical note reduces the need for repetition of background material by individual study papers, which allows these papers to focus on specific analyses and the presentation of the results. It also allows the reader to access all relevant background material in a single location rather than spread out over several papers. Because of this design, this technical note should not be viewed as a stand-alone scientific paper as it does not contain any results but rather as laying the groundwork for subsequent scientific papers contributed by Activity 1 modelling groups to the AQMEII4 special issue.

## 2 AQMEII4 Activity 1 description

Activity 1 like the previous phases of AQMEII includes the evaluation of regional air quality model simulation on the NA, EU, or both domains for at least a 1-year period. Prior to describing the requested output that pertains strictly to dry deposition, we briefly summarize the modelling periods and domains, common inputs, and standard concentration, meteorology, and wet deposition outputs for Activity 1 in this section.

### 2.1 Modelling periods and domains

For AQMEII4 Activity 1 the air quality community listed in Table 1 has been asked to perform two annual simulations of the air quality over NA and/or EU.

Specifically, the years of interest in AQMEII4 are North America – 2010 and 2016 and Europe – 2009 and 2010. The NA years were selected due to their policy relevance; the years 2010 and 2016 have featured in policy-relevant emissions scenario simulations by governments on the continent. In the case of Europe, the years illustrated a marked difference in meteorological signatures between the 2 years, hence providing a gauge of the impact of meteorological variability on deposition. Modelling multiple years also allows the investigation of the variability of impacts of emission policies and weather conditions on deposition patterns.

**Figure 1.**
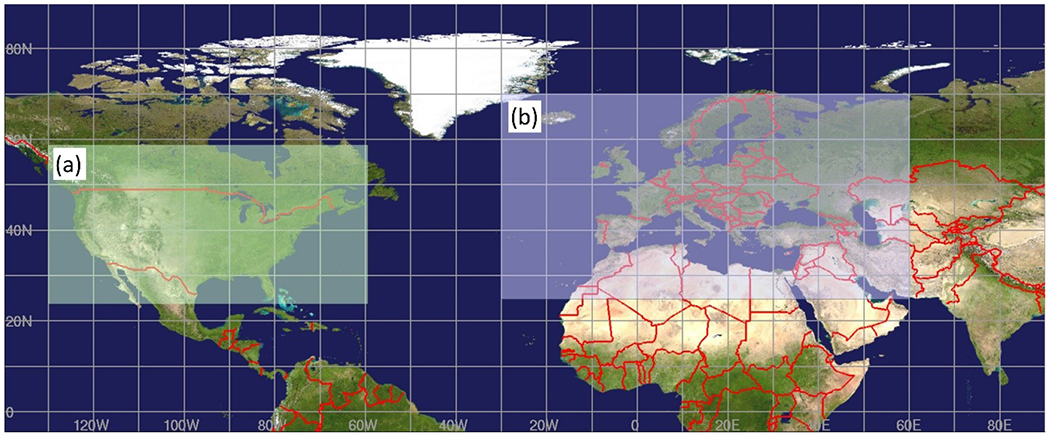
AQMEII4 North American (**a**) and European (**b**) 0.125° grid cell size common latitude–longitude comparison domains.

All modelling groups carried out simulations on their own grid projections. These “native grid” simulations were interpolated to a common 0.125° × 0.125° latitude–longitude (Fig. 1) grid over each continent to allow direct comparison of gridded model data:

- NA: 23.5° N↔58.5° N, 130° W↔59.5° W,
- EU: 25° N↔70° N, 30° W↔60° E.

Modelling groups are expected to perform their simulations on a grid with comparable to higher horizontal resolution as these reported grids. For the interpolation of model results from the native modelling grid to the common analysis grid, a mass-conserving method was recommended for concentrations and fluxes, and the nearest-neighbour method was recommended for diagnostic variables.

### 2.2 Model inputs shared by all participants

Air quality models require input fields for meteorology, emissions, and chemical boundary conditions; differences in each of these fields lead to differences in model results. All AQMEII exercises have considered the driving meteorology to be an integral part of each participating model (for online models, such as studied under AQMEII-2, chemistry and meteorology are inseparable, since both are included in the same modelling platform) and have therefore not attempted to harmonize meteorological fields across participants. However, variations caused by different emissions and chemical boundary conditions are removed in all AQMEII phases by requiring all participating models to use a common set of emissions and lateral chemical boundary conditions (Galmarini et al., 2012, 2015, 2017). Note that due to their dependence on model-specific LULC and meteorology, biogenic emissions are not prescribed and are generated by each group. For AQMEII4, the common model inputs were prepared as follows.

#### 2.2.1 Anthropogenic emissions

Emissions for anthropogenic sources over NA were prepared from US, Canadian, and Mexican inventory data using the emissions processing approach developed for U.S. EPA “emission modelling platforms” (EMPs). An EMP includes not only the underlying point source, county, or province level inventory data but also controls the temporal and spatial allocation and chemical speciation of these inventories. For 2010, the processing was based on the “2011v6.3 EMP” (https://www.epa.gov/air-emissions-modeling/2011-version-63-platform, last access: 7 October 2021). Year-specific adjustments for 2010 were made to the EMP for several sectors (e.g. electric generating units, mobile sources, and residential wood combustion), and Canadian emissions were based on a 2010 inventory rather than the 2013 inventory projected to 2011 used in the EMP. For 2016, the processing was based on the “2016beta EMP” (https://www.epa.gov/air-emissions-modeling/2016v72-beta-and-regional-haze-platform, last access: 7 October 2021), which is documented at http://views.cira.colostate.edu/wiki/wiki/10197, last access: 7 October 2021. These EMPs were used by the U.S. EPA to generate eight different hourly files of speciated emissions for each day in 2010 (one gridded file with low-level emissions and files with elevated sources from seven different sectors) and nine different hourly speciated files for each day in 2016 (one gridded file with low-level emissions and files with elevated sources from eight different sectors), which were then shared with all participants. Speciation was performed for both the CB6R3 and SAPRC07 mechanisms to provide flexibility to participants to map emissions to the chemical mechanism used in their model. The same data were used by Environment and Climate Change Canada to generate day-specific emissions for the GEM-MACH air quality model, for the ADOMII mechanism used within that model. Annual gridded anthropogenic emissions using the Standard Nomenclature for Air Pollution (SNAP) sector classification scheme were prepared over EU by TNO for 2009 and 2010 as part of the MACC-III project (Kuenen et al., 2015) and were provided to EU modelling groups along with reference temporal allocation and speciation profiles. If necessary, EU modelling groups used other emission datasets available to them to fill in emissions near the edges of their modelling domains if their modelling domains extended beyond the area covered by the MACC-III emissions provided by TNO.

#### 2.2.2 Forest fire emissions

The forest fire emissions over NA for 2010 were a combination of emissions over the US included in the “2011v6.3” EMP and emissions over Canada provided by Environment and Climate Change Canada (ECCC; Chen et al., 2013) while 2016 forest fire emissions over both the US and Canada were obtained from the “2016 beta” EMP. Data distributed to modelling groups included both the mass of emissions of criteria air contaminants (speciated into the gases of the gas-phase chemistry mechanisms noted above) and the parameters necessary to compute plume rise using a prescribed plume rise algorithm based on the large stack plume rise formula of Briggs (Briggs, 1971, 1972). While different modelling platforms often have their own approaches for estimating forest fire emissions, particularly in an operational context, as was the case for anthropogenic emissions, this unified approach was adopted in order to reduce the variability in model performance associated with emissions inputs. Forest fire emissions for 2009 and 2010 over EU were provided by the Finnish Meteorological Institute and were developed using the IS4FIRESv2 methodology described in Soares et al. (2015). These emissions were vertically allocated to eight layers with heights ranging from 50 to 6200 m, with individual groups re-allocating the resulting mass to their own vertical discretization.

#### 2.2.3 NO emissions from lightning

Although previous phases of AQMEII did not consider NO emissions from lightning, these emissions were included in the current phase due to their impact on nitrogen deposition fluxes. To provide a unified forcing from this source across all models, the emissions were based on the GEIA monthly climatology (Price et al., 1997) rather than in-line parameterizations based on meteorological fields implemented in some but not all participating models. Although using climatological lightning does not capture the linkage between modelled meteorology and NO emission from lightning, this approach ensures that the bulk effects are included in all modelling systems and streamlines the interpretation of the modelling results by removing a potential difference in emissions input. The monthly climatological values were allocated diurnally based on Table 2 in Blakeslee et al. (2014) and distributed to participating groups as two-dimensional files. Groups were then asked to allocate these emissions to their specific vertical grid based on Table 2 of Ott et al. (2010), using the tropical profiles for land and water (or an average of the two) for grid cells with latitudes below 23.5° N, the subtropical profile for grid cells with latitudes between 23.5 and 40° N, and the mid-latitude profile for grid cells with latitudes >40° N.

#### 2.2.4 Chemical boundary conditions

Concentrations of the 33 longer-lived trace gas and aerosol species listed in Table 2 were provided by the European Centre for Medium-Range Weather Forecasts (ECMWF) for the two continents and for the modelled time periods so that participants could prepare initial and boundary conditions for their regional-scale modelling domains. The concentration fields were based on the Copernicus Atmospheric Monitoring Service (CAMS) EAC4 reanalysis product (Inness et al., 2019) and were provided every 3 h on a 0.75° × 0.75° grid with 54 vertical levels from the surface to 2 hPa. The vertical grid structure varied in both resolution and vertical extent across models, and individual participants were responsible for interpolating the CAMS fields to their horizontal and vertical grid structure. The CAMS species were matched by participants to their own internal model speciation (and, in the case of the particulate matter emissions, to the particle size distribution of their own models).

### 2.3 Standard model outputs

We distinguish here between model output similar in scope and intent to previous ensemble model comparisons in past phases of AQMEII (i.e. “standard model outputs”) and the detailed diagnostic outputs reported under AQMEII4. The standard output requested from all participating models comes in two major forms: as hourly gridded surface concentrations and meteorological variables on the common grids described earlier and as model values extracted at monitoring network station locations. Tables A1–A3 of Appendix A list the variables requested for gas- and particle-phase species, meteorology, and grid-scale deposition fluxes. The meteorological variables have been extended considerably compared to past phases of AQMEII, to include more parameters that describe the planetary boundary layer. The gridded fields of integrated emissions were also requested as output, to be used to check that the right amounts of masses were inputted into the models.

A list of all available surface monitoring locations in both continents for concentrations of gas- and particle-phase species, precipitation chemistry, and meteorology was distributed to the AQMEII4 participants who are expected to produce model results for all species presented in Appendix A for the grid location closest to the monitor or interpolated to the monitoring. In particular, we note that the analysis of wet deposition in AQMEII4 will rely on the precipitation and wet deposition flux variables listed in Table A3. Note that the units of nitrogen and sulfur deposition in Table A3 are “equivalents” per hectare per year, where the equivalent refers to the product of moles and the oxidized charge associated with the deposited species. All species depositing sulfur are assumed to have a charge of 2, and all species depositing nitrogen we assumed to have a charge of 1. These units are used in the calculation of exceedances of critical loads, where the annual charge balance and flux of charge to ecosystems is used to estimate potential ecosystem impacts. For more information on the routine monitoring networks used in AQMEII, please refer to Galmarini et al. (2012, 2015, 2017).

## 3 Strategy for the diagnostic intercomparison of dry deposition from different grid-based models

Analysis of dry deposition is the focus of AQMEII4. In particular, AQMEII4 intends to go beyond an operational evaluation of ambient concentrations and comparison of total deposition across models because this approach does not provide enough information to determine the causes of different deposition totals among regional models. The novelty of AQMEII4 is that we request additional and very detailed diagnostic-evaluation outputs related to dry depositional from all of the models. With these very detailed outputs, we can compare the important elements of the model machinery and understand model differences.

Many regional models use the Wesely (1989) dry deposition scheme, but several variants have been developed and implemented with different levels of sophistication. Dry deposition schemes are mostly resistance frameworks – by framework, we mean the structure of the scheme with respect to how processes relate to one another – and all of the regional models in AQMEII4 use resistance frameworks for dry deposition. Resistance frameworks are based on the representation of series and parallel resistors in electrical circuits. Differences in resistance frameworks across regional models imply that comparing a given process among the regional models is not straightforward. Thus, diagnostic variables that account for differences in resistance frameworks need to be reported. Below, we present the strategy devised to reduce any dry deposition scheme to the essential set of comparable variables regardless of the differences in the frameworks of the schemes that generated them.

We start with a description of the Wesely (1989) resistance framework, one of the earliest literature examples of a resistance framework for dry deposition and arguably the most popular dry deposition scheme, and follow with both generic and specific examples of other resistance frameworks as a guide to the AQMEII4 output protocol. The components of the deposition velocity are process-based resistances (units are s cm^−1^) that impede the transfer of mass to a variety of surfaces. Resistances are added in series for processes operating on the same depositional pathway, and in parallel when multiple surfaces for dry deposition exist. In the original Wesely (1989) scheme, four deposition pathways were used: soil, “lower canopy and exposed surfaces”, leaf cuticles, and plant stomata. Gases are first impeded by an aerodynamic resistance to deposition (*r*_a_), second impeded by a quasi-laminar sublayer resistance (*r*_b_), and third impeded by a bulk surface resistance term (*r*_c_) composed of a parallel summation of the resistances associated with each pathway. The three impedances to deposition are added into a total resistance, the inverse of which is the deposition velocity of the gas (units = cm s^−1^):

(1) $$v_{d}={\left(r_{a}+r_{b}+r_{c}\right)}^{-1}.$$

The bulk surface resistance (*r*_c_) in Wesely (1989) follows

(2) $$r_{c}=({\left(r_{s}+r_{m}\right)}^{-1}+{\left(r_{lu}\right)}^{-1}+{\left(r_{dc}+r_{cl}\right)}^{-1}{+{\left(r_{ac}+r_{gs}\right)}^{-1})}^{-1}.$$

**Figure 2.**
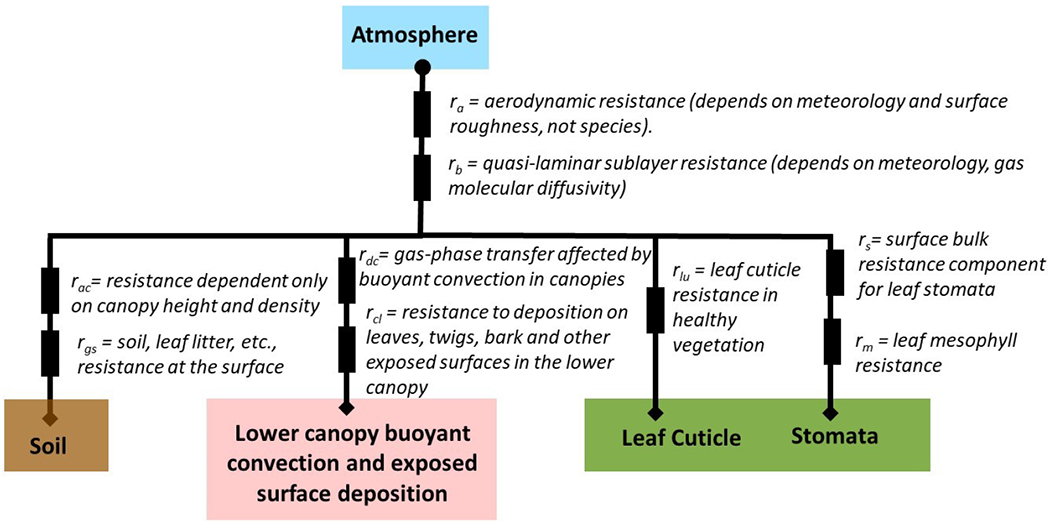
Schematic of the resistance framework for gas-phase dry deposition for the Wesely (1989) scheme. Circles and diamonds show where ozone concentration is needed as input for a given framework. At the diamonds, the ozone concentration is assumed to be zero. Rectangles indicate resistances.

The component resistances used in *r*_c_ are defined in Fig. 2, which is a schematic of the Wesely (1989) resistance framework.

Work subsequent to Wesely (1989) also uses the resistance approach, but sometimes with considerable variation in the resistance framework, the number of surfaces to which dry deposition occurs, and/or the processes represented by individual resistances. Several motivating factors likely led to the development of a diversity of resistance frameworks. In the intervening years subsequent to Wesely’s introduction of the resistance framework concept, new measurement capabilities (for higher-time-resolution information, for greater chemical speciation, higher-precision measurements) allowed the original algorithms to be tested and modified. Developments in plant physiology understanding have also resulted in improved stomatal resistance parameterizations. Examples include the observation-based introduction of bidirectional fluxes for ammonia gas and improved understanding of the role of CO_2_ fluxes in the deposition of other gases. Also, some divergence in approaches is likely due to algorithm developments having been made in the context of specific regional models – each of which encompasses a diverse range of process representation algorithms, vertical resolutions, horizontal resolutions, etc. An algorithm which provided good performance relative to surface concentration observations within the context of one regional model thus may not have resulted in as good of performance in another model, further spurring *model-specific* development. These factors have resulted in the variety of approaches for gas-phase deposition in current regional models and provide the part of the motivation for this first attempt at cross-comparing the results of the models’ deposition algorithms in detail – to show and explain the causes for these differences.

**Figure 3.**
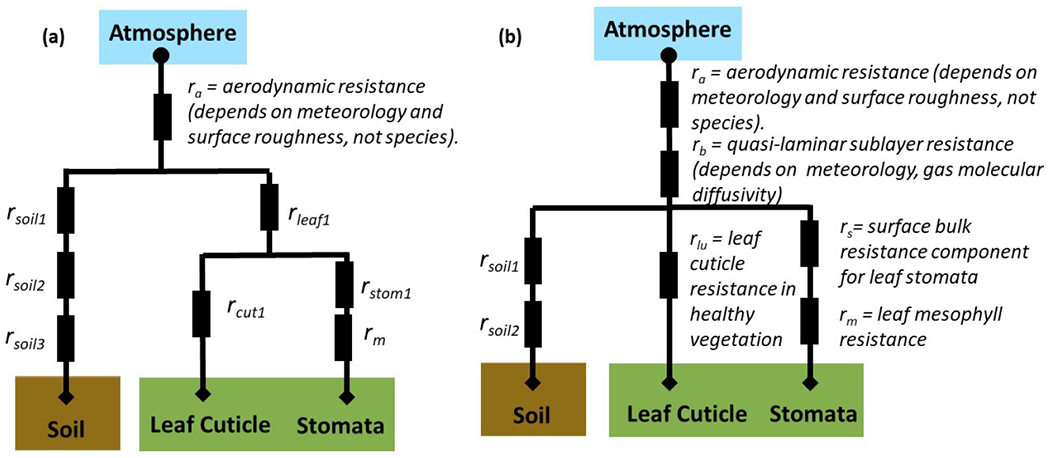
Two generic deposition resistance examples.

Schematics of resistance frameworks as two generic examples are shown in Fig. 3. In these examples, the Wesely (1989) deposition pathway for “lower canopy buoyancy and exposed surfaces” deposition is not included. The example of Fig. 3a also lacks a quasi-laminar sublayer resistance *r*_b_ applied across all surface types. Instead, surface-specific quasi-laminar sublayer resistances are used: *r*_soil2_ for soil and *r*_leaf1_ for leaves. The examples in Fig. 3 demonstrate two ways in which the resistance framework has been adapted from Wesely (1989). In general, the diversity in resistance frameworks across models complicates model intercomparison of individual resistances.

When there are differences in resistance frameworks across models, the deposition pathways may be compared across models using a construct we will refer to here as *effective conductance* (Paulot et al., 2018; Clifton et al., 2020b). While generally a conductance is simply the inverse of a resistance, an *effective* conductance is the contribution of a given depositional pathway to the deposition velocity, expressed in the same units as the deposition velocity. The sum of the effective conductances for all deposition pathways is the deposition velocity. The effective conductances of the soil (*E*_SOIL_), lower canopy (*E*_LCAN_), cuticle (*E*_CUT_), and stomata (*E*_STOM_) branches specifically for Wesely (1989) are given by^1^:

(3) $$E_{\text{SOIL}}=\left(\frac{{\left(r_{ac}+r_{gs}\right)}^{-1}}{{\left(r_{s}+r_{m}\right)}^{-1}+{\left(r_{lu}\right)}^{-1}+{\left(r_{dc}+r_{cl}\right)}^{-1}+{\left(r_{ac}+r_{gs}\right)}^{-1}}\right)v_{d}$$

(4) $$E_{\text{LCAN}}=\left(\frac{{\left(r_{dc}+r_{cl}\right)}^{-1}}{{\left(r_{s}+r_{m}\right)}^{-1}+{\left(r_{lu}\right)}^{-1}+{\left(r_{dc}+r_{cl}\right)}^{-1}+{\left(r_{ac}+r_{gs}\right)}^{-1}}\right)v_{d}$$

(5) $$E_{\text{CUT}}=\left(\frac{{\left(r_{lu}\right)}^{-1}}{{\left(r_{s}+r_{m}\right)}^{-1}+{\left(r_{lu}\right)}^{-1}+{\left(r_{dc}+r_{cl}\right)}^{-1}+{\left(r_{ac}+r_{gs}\right)}^{-1}}\right)v_{d}$$

(6) $$E_{\text{STOM}}=\left(\frac{{\left(r_{s}+r_{m}\right)}^{-1}}{{\left(r_{s}+r_{m}\right)}^{-1}+{\left(r_{lu}\right)}^{-1}+{\left(r_{dc}+r_{cl}\right)}^{-1}+{\left(r_{ac}+r_{gs}\right)}^{-1}}\right)v_{d}$$

The denominator in each of Eqs. (3) to (6) is the inverse of the bulk surface resistance *r*_c_ and the numerators are the inverses of the resistances associated with each pathway in *r*_c_. We emphasize that the calculation of the effective conductances depends on the resistance framework used; Eqs. (3) to (6) are specific to Wesely (1989) and require modification for other resistance frameworks, and we provide examples of formulae for these terms for other frameworks, in Sect. 4.1 and Appendix B. Calculation of the effective conductances requires either archiving all component resistances in a given framework and subsequent post-processing or their online calculation.

For any given model, effective conductances are an invaluable tool for determining the extent to which each pathway impacts dry deposition velocity, and which deposition pathways drive spatiotemporal variability in dry deposition velocity. Key for AQMEII4, the effective conductances allow a cross-comparison of the main deposition pathways across different resistance frameworks. The primary terms of comparison for dry deposition schemes in AQMEII4 are thus the effective conductances. In addition, given that many models’ resistance frameworks follow Wesely (1989), we also request those individual resistance terms held in common by most models to allow exact comparisons of individual processes which may influence or control a given pathway. These resistances include

1. a term for the aerodynamic resistance, *r*_a_;
2. a term for the bulk resistance to deposition associated with surfaces, *r*_c_;
3. a term or series addition set of terms describing the stomatal resistance, *r*_s_;
4. a term or series addition set of terms describing the mesophyll resistance, *r*_m_;
5. a term or series addition set of terms describing the cuticle resistance, *r*_c_;
6. terms to describe quasi-laminar sublayer resistance, *r*_b_;
7. a term to describe within-canopy buoyant convection, *r*_dc_.

With regards to (6), the implementation of quasi-laminar sublayer resistance (*r*_b_ in Wesely, 1989) tends to differ among models. Some models use the Wesely (1989) concept of a pathway-independent quasi-laminar sublayer resistance. Others use quasi-laminar sublayer resistances as pathway-dependent (e.g. Fig. 2a, where the *r*_soil2_ and *r*_leaf1_ represent quasi-laminar sublayer resistances for soil and leaf pathways, respectively). The quasi-laminar sublayer resistance is thus reported in AQMEII4 for each pathway, with the models for which the term is independent of pathway reporting the same value for each pathway. Pathway-dependent quasi-laminar sublayer resistances are to be reported as “not present” only if the given pathway does not exist in the framework.

**Figure B2.**
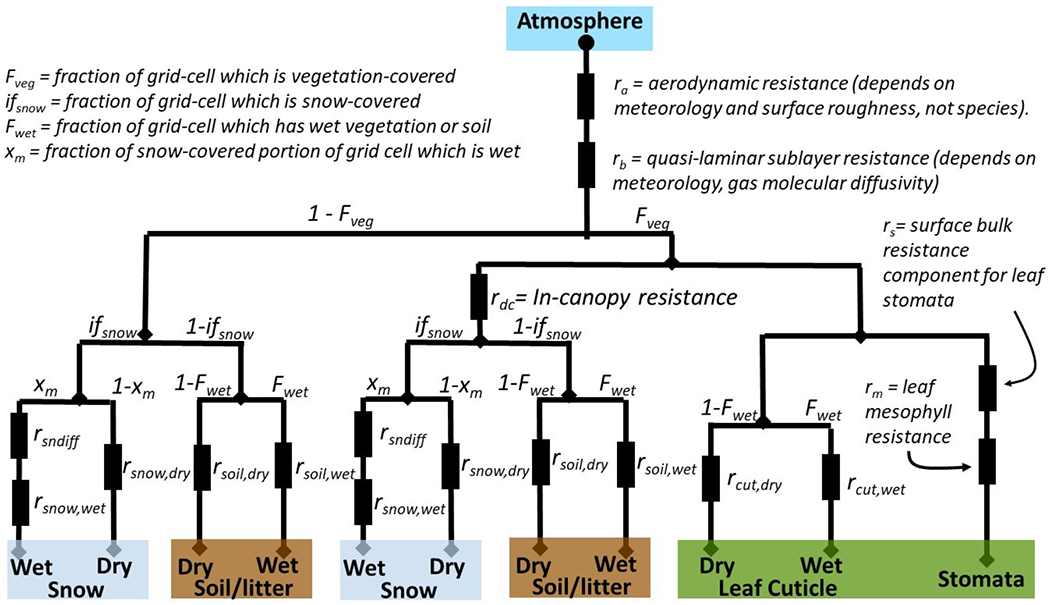
Resistance diagram for the U.S. EPA CMAQ model with the M3DRY deposition option.

**Figure B3.**
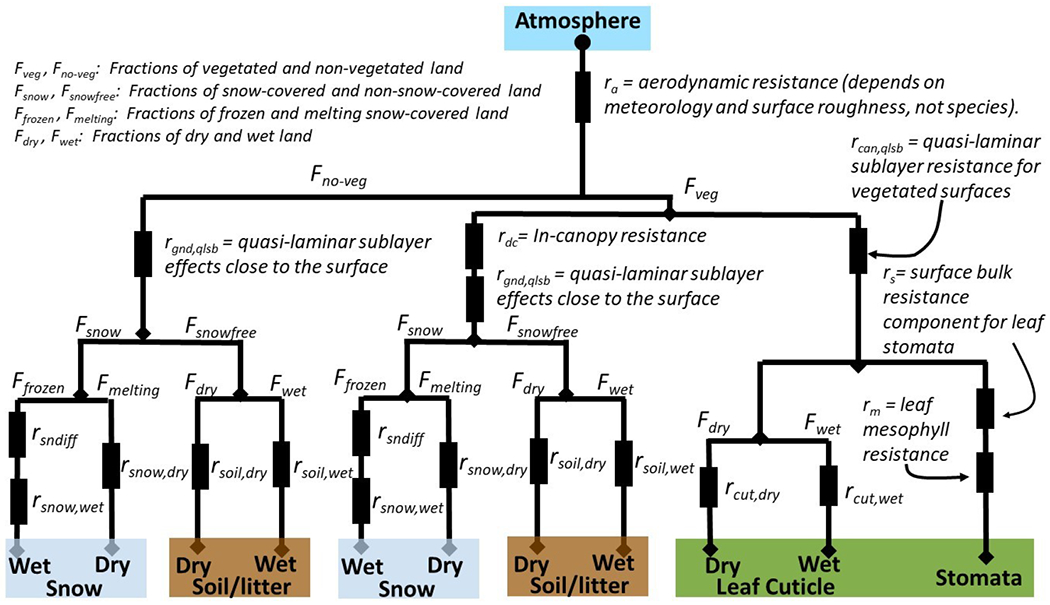
Resistance diagram for the U.S. EPA CMAQ model with the STAGE deposition option. Note that this is an extension of the Massad et al. (2010) and Nemitz et al. (2001) resistance model in the CMAQ modelling framework.

**Figure B4.**
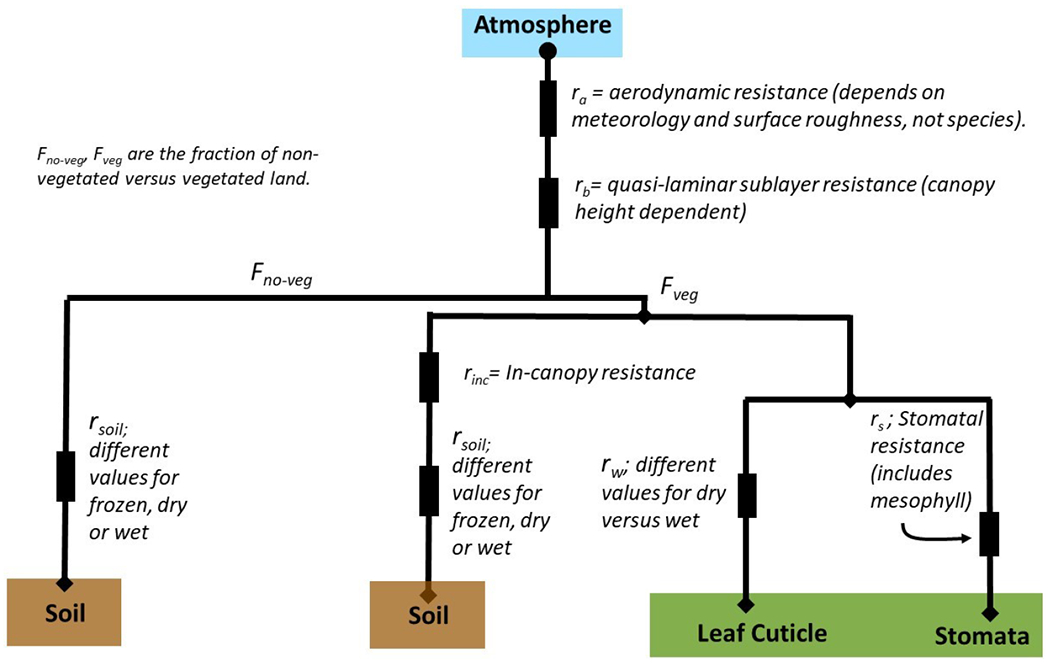
Resistance diagram for the dry deposition scheme implemented in LOTOS EUROS.

Note that models that include a single deposition pathway to soil that incorporates *r*_dc_ are requested to report that pathway as “lower canopy” not “soil”. For example, the LOTOS-EUROS dry deposition scheme (Fig. B4) reports the effective conductance calculated for the soil pathway as *E*_LCAN_ due to the presence of the in-canopy resistance term in this pathway. In contrast, the CMAQ-M3DRY and CMAQ-STAGE dry deposition schemes (Figs. B2 and B3) have two separate pathways for deposition to soil, one for vegetation-covered soil and one for bare soil. Due to the inclusion of the in-canopy convective resistance in the computations for vegetation-covered soil, the effective conductance for that pathway is reported as *E*_LCAN_, while the effective conductance for the bare soil pathway should be reported as *E*_SOIL_.

Specific resistance terms for the soil deposition pathway and the lower canopy pathway have not been requested because the resistance frameworks for these pathways vary considerably across models, and therefore specific resistance terms are not easily comparable. For example, Wesely (1989) used a single term for the soil resistance (Fig. 1) while other models may use two or three resistances related to dry deposition to soil only and added in series (Fig. 2).

In addition to the effective conductances, another set of diagnostic fields is calculated during post processing: the time-aggregated fractional mass (or charge equivalent) *flux* transferred to the surface via each of the four deposition pathways (hereinafter, *effective flux*). The effective flux is calculated on an hourly basis prior to conversion to AQMEII4 time-aggregated gridded and station data using ENFORM and is the product of the hourly effective conductances, dry deposition mass fluxes, and inverses of the deposition velocity. Effective *conductances* provide an estimate of the importance of each pathway towards the deposition velocity. However, since the flux depends on the deposition velocity and the near-surface air concentration, which both vary on hourly timescales, estimating the aggregate importance of each deposition pathway towards the flux requires calculating the effective flux before time aggregation.

**Figure 4.**
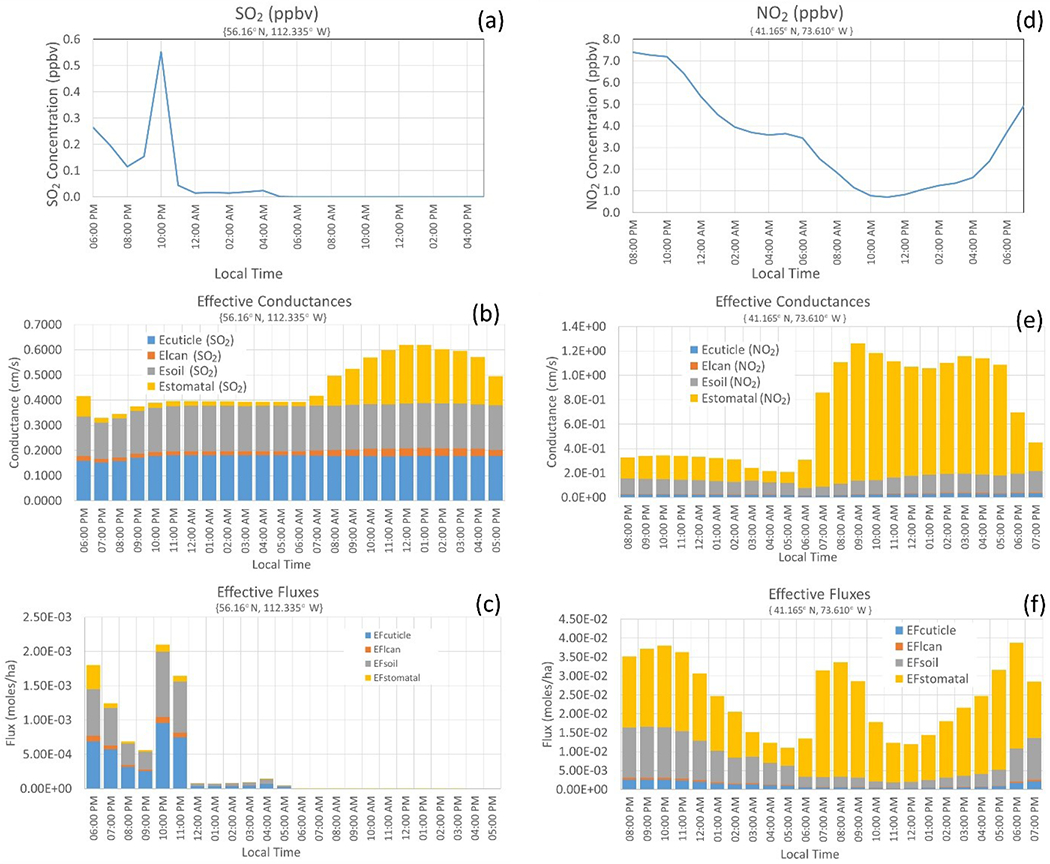
Two examples of diurnal variations in concentrations (**a, d**), effective conductances (**b, e**), and effective fluxes (**c, f**) for SO_2_ (left column) and NO_2_ (right column).

Figure 4 provides an example of the different yet complementary information resulting from effective conductances and effective fluxes, showing hourly SO_2_ concentrations, effective conductances, and effective fluxes for a boreal forest impacted by a large industrial SO_2_ stack source and hourly NO_2_ concentrations, effective conductances, and effective fluxes for a location to the north-east of New York City. In both cases, high concentrations of the pollutant gas (Fig. 4a, d) occur at night, while the deposition velocity, due to the stomatal pathway (Fig. 4b, e), maximizes during the day. As a result of the low daytime concentrations, the effective fluxes for SO_2_ (Fig. 4c) show a relatively minor contribution of the stomatal pathway to the deposited mass despite the major contribution of the stomatal pathway to the daytime deposition velocity. As the result of high night and morning concentrations, the effective fluxes for NO_2_ (Fig. 4f) show separate day and night peaks of about equal magnitude, with the stomatal pathway dominating daytime values and roughly equivalent contributions from stomatal and soil pathways at night.

Also with reference to Fig. 4, it should be noted that the effective conductances and effective fluxes show the relative contributions of the pathway towards the total deposition or the total flux at any given time. It should also be noted that the net surface resistance appearing in the denominator of these terms may drive the time variation. For example, the soil effective conductance of Fig. 4e minimizes at 6:00 LT – however, the factors contributing to the soil pathway itself for the model used in this example (see Appendix Table B1) are relatively time-invariant (seasonally varying). The temporal variation is driven by hourly variation in the stomatal term and hence the relative importance of the soil conductance varies with time in Fig. 4e.

We also consider that dry deposition strongly depends on LULC type, and different models use unique LULC databases. We thus request LULC-specific variables along with the fractional areal coverage for each LULC type, which allows quantification of not only the impacts of different LULC-specific processes and parameters on dry deposition, but also the impacts of different LULC databases. “Generic” AQMEII4 LULC types were devised due to the use of a wide variety of LULC databases across air quality models, in terms of both the source of the data and the number of LULC types employed. The AQMEII4 LULC types listed in Table 2 are broad LULC types into which the model-specific LULC types could be aggregated, to allow intercomparison between models. Study participants aggregated their LULC-model-specific diagnostic outputs to the set of common AQMEII4 LULC types using the fractional representation of each native LULC type contributing to the AQMEII4 type within each grid cell. Generic AQMEII4 LULC types were constructed after analysis of the LULC schemes in the participating models. A suggested mapping between model and AQMEII4 LULC types was provided to participants, along with the instruction that the mapping actually employed should be reported. The grid cell fractions of both the native model LULC types and the resulting fractions of AQMEII4 LULC types were reported by participants. Note that there is a large variety in number and therefore types of LULC across models, and thus each of the generic types represents a rather broad range of LULCs.

We also note that the mapping of LULC types from the individual model land use classifications to the AQMEII4 land use classifications is an unavoidable source of uncertainty in the land-use specific diagnostics. The 15 AQMEII4 land use types themselves were based on a survey of land-use classifications used in 17 regional models. For example, while “Herbaceous” is available as an AQMEII4 land use category, its intent is for use for moors and heathlands, while AQMEII4 land use category “Wetlands” encompasses wetlands which are diversely described in individual model land use categories such as herbaceous, wooded, and permanent wetlands, as well as swamps and peatbogs. However, some categories were included most models (e.g. evergreen needleleaf forest, deciduous broadleaf forest, snow and ice, mixed forest, usually taken as a combination of needleleaf and deciduous forests), while others could easily be classified according to the broader landscape type of which they were a member (e.g. different types of tundra were recommended to be classified as the AQMEII4 Tundra classification). Both the AQMEII4 and “native model” land use types were reported by participants – with the aim of using both sets of information to determine the extent to which land use database variation may be a factor in estimating deposition velocities and to provide information on specific land use types used by specific models when these differences appear to be large.

For AQMEII4, the terms listed in Table 4 were reported for SO_2_, NO_2_, NO, HNO_3_, NH_3_, PAN, HNO_4_, N_2_O_5_, organic nitrates, O_3_, H_2_O_2_, and HCHO, both as a function of the 16 generic AQMEII4 LULC types (Table 3) and for the net grid-scale calculation for each grid cell and/or receptor. Models employing bidirectional flux algorithms for the dry deposition of atmospheric NH_3_ reported a different set of terms, given in Sect. 4.2.

Table 4 summarizes the diagnostic variables related to gaseous dry deposition reported by all participants, the variable names as described in the AQMEII4 technical specification documents (TSDs), and a description of each variable. Equations (2) through (6) and the related text describe the terms specifically for the resistance framework of Wesely (1989); additional examples for participating models’ resistance frameworks are provided in the Appendix tables and figures.

The presence of surface wetness or snow is incorporated into the effective conductance, effective flux, and component resistances. In other words, separate component resistances or effective conductances and fluxes for snow-covered or wet surfaces were not reported. In order to compare the impacts of the different models’ predictions regarding snow cover or wetness, additional diagnostic variables were requested to describe surface state (e.g. fractional snow cover and either the values of binary wet–dry conditions or fractions in surface wetness).

Gridded dry deposition diagnostic variables were archived as hourly values for the native LULC types and then converted to the generic AQMEII4 LULC types during postprocessing. The ENFORM Fortran code provided to all participants was used to convert gridded fields from the hourly values to temporal aggregations of the hourly values. Hourly diagnostics were converted to “monthly median diurnal” values using ENFORM by taking the medians of all values for a given UTC hour in a given month, thus reducing 8760 hourly values for each year to 288 values (24 h × 12 months). The use of monthly median diurnal values is motivated by the need to reduce the amount of data to be transferred and analysed on a single server (despite this aggregation, each year of gridded model output requires up to 200 Gb of storage), while preserving the key aspects of diurnal and seasonal variations.

The use of a median rather than an arithmetic mean for AQMEII4 diagnostic time aggregation resulted from consideration of the manner in which different dry deposition algorithms deal with pathways that effectively shut down under certain conditions. For example, some algorithms employ an upper-limit resistance to represent conditions under which the pathway transmits little mass to the surface (e.g. nighttime stomatal resistances may be set to very large values). Others simply use code branching to prevent a pathway from contributing to *r*_c_ (e.g. the entire stomatal pathway is removed from r_c_ at night). Others employ different resistance frameworks for different conditions (e.g. to account for snow-covered surfaces). However, the AQMEII4 protocol requires participants to submit “missing values” as a specific code (−9) in order to allow filtering of valid from invalid data during time aggregation. An algorithm removing a pathway may thus have a different number of valid values from an algorithm employing a large resistance. Similarly, a seasonal transition where the resistance network changes depending on whether a surface is snow-covered becomes difficult to interpret in a time average, whereas valid time-median values allow for a more meaningful comparison.

For example, if only 20 % of the resistances at 14:00 LT in a given month and grid cell are snow covered, then the monthly median for 14:00 LT would represent values typical of snow-free conditions, for both models representing resistances under snow-covered conditions as missing and models representing them as large values. Thus, the monthly median comparison represents the most common conditions encountered during the month for both models. On the other hand, while the monthly average resistance for 14:00 LT represents snow-free conditions for the model that treats snow-covered hours as missing, the monthly average for the model that represents snow-covered conditions as a large value is not meaningful and complicates inter-model comparison.

Monthly median diurnal values capture both seasonal and diurnal variations in the archived fields and allow comparisons between algorithms shutting off a pathway by removing the pathway and algorithms shutting off a pathway with high resistance values. Note that the same data completeness criterion used for comparing simulated and observed ambient concentrations was employed here for the construction of the median values. Specifically, more than 75 % of the values within a month were required for a median to be constructed.

## 4 More example calculations of AQMEII4 dry deposition variables

### 4.1 Variations on the Wesely (1989) resistance framework

For the sake of clarity, we provide examples of how specific dry deposition schemes can be reduced to the common set of variables described above. The generic schemes presented in Fig. 2a, b along with the Nemitz et al. (2001) bidirectional scheme for NH_3_ have been selected as examples here, while Appendix B provides additional examples for specific schemes implemented in participating models. The AQMEII4 protocol and these specific examples provide a standard form of representing key aspects of dry deposition schemes, which may be adopted by similar activities or initiatives in the future. Note that some of these example algorithms do not have a separate resistance for lower canopy buoyant convection or a deposition pathway to the lower canopy and exposed surfaces; hence the associated effective conductance (ECOND-LCAN) and resistance (RES-CONV and RES-QLLC) terms are not reported.

### 4.2 Bidirectional fluxes of ammonia – a special case

**Figure 5.**
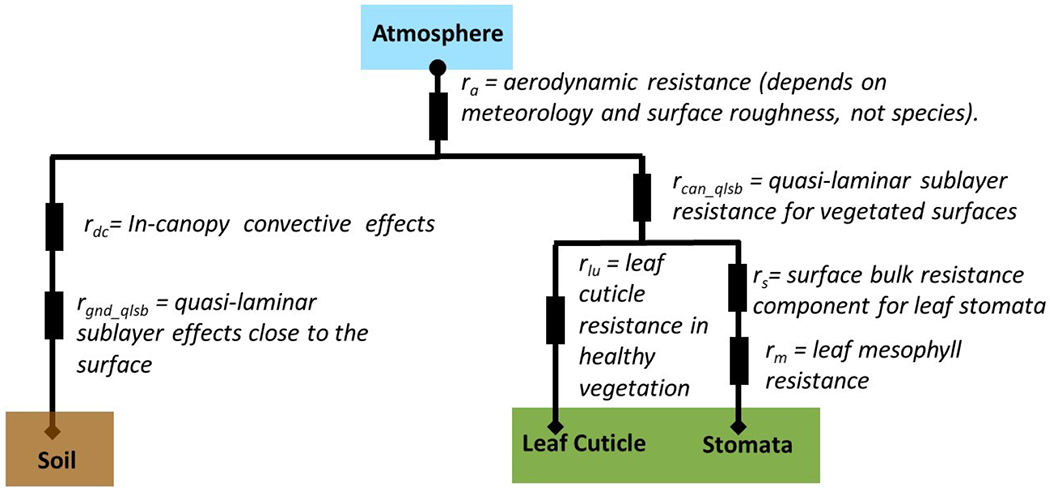
Nemitz bidirectional flux model for NH_3_.

Some models make use of the concepts of bidirectional fluxes when describing ammonia gas transfer from and to surfaces. In the bidirectional flux paradigm, the difference between the ambient gas concentrations and near-surface (compensation point) concentration is used to determine the direction of the flux: if the ambient air concentration is greater than the compensation point concentration, the flux is downward (i.e. deposition occurs) while in the reverse case the flux is upward (i.e. the emission of ammonia previously stored in the surfaces takes place). The algorithms used in the subset of models employing ammonia bidirectional fluxes were examined, in order to determine common terms that could be used for points of comparison across the algorithms. As an example, we present the bidirectional flux model of Nemitz et al. (2001) below (Fig. 5 and Table 7), used within CMAQ to represent bidirectional ammonia gas fluxes. In addition, we also include a comparison of two ammonia bidirectional flux calculations in Appendix C.

The bidirectional flux algorithms were analysed as a separate case, with the result that a revised and smaller number of variables were reported for the specific case of ammonia bidirectional fluxes than for other gases, focusing on the compensation point concentrations as diagnostics for the cross-comparison of these algorithms. The reported variables in this case are ammonia’s aerodynamic resistance, its net surface resistance, and three compensation point concentrations, for stomata and ground and net compensation points, respectively. These specific parameters for ammonia bidirectional fluxes appear in Table 7, and a detailed comparison of two representative bidirectional ammonia algorithms is presented in Appendix C.

In this example, note that the branch containing the “soil” term has been designated as the lower canopy pathway, due to the presence of the canopy buoyant convection term *r*_dc_ (i.e. closest analogy to Wesely’s setup is to have the pathway involving deposition to “soil” pathway designated as a “lower canopy” pathway).

## 5 Conclusions

The fourth phase of the Air Quality Model International Initiative has been introduced. The focus of this phase is on wet and especially dry deposition. The necessity of tackling this subject in a diagnostic way prompted us to divide the initiative into two activities, one dedicated to the evaluation of the process as described by four-dimensional air quality regional-scale models and the second dealing specifically with evaluating ozone dry deposition calculated by “single-point model” versions of the dry deposition modules used in the regional-scale models with a collection of ozone flux measurements. Here, the organization of Activity 1 has been formally introduced, whereas Activity 2 will be described in separate AQMEII4 special issue publications. In addition to presenting the standard and common input data and the way in which standard output is expected, we also presented the way in which the very diverse representations of dry deposition in participating models have been reduced to a common representation that will facilitate model inter-comparison. The essence of the adopted methodology is the transformation of individual resistances into effective conductances and effective fluxes, which represent the importance of deposition pathways held in common across the models to the total deposition velocity and flux. Resistances held in common across different modelling frameworks were also reported, to allow comparisons at the sub-pathway level, where possible. Thus, regardless of the level of sophistication of the resistance framework, one can meaningfully intercompare the results produced by different models.

## Figures and Tables

**Table 1.** Participating institutes, model names, and cases simulated.

Group/institution	Modelling system	Model domains	References
Leibniz Institute for Tropospheric Research (TROPOS), Germany	COSMO/MUSCAT	EU	Wolke et al. (2012) Chen et al. (2018)
Environment and Climate Change Canada (ECCC), Canada	GEM/MACH (three different model configurations)	NA	Makar et al. (2021) Makar et al. (2018) Makar et al. (2017) Moran et al. (2010)
Technical University of Madrid (UPM), Spain	WRF-Chem	EU and NA	Grell et al. (2005)
Netherlands Organization for Applied Scientific Research (TNO), the Netherlands	LOTOS/EUROS	EU	Manders et al. (2017)
Institute for Advanced Sustainability Studies (IASS), Germany	WRF-Chem	EU and NA	Grell et al. (2005) Fast et al. (2006)
U.S. Environmental Protection Agency, USA	WRF/CMAQ (two different model configurations)	NA	U.S. Environmental Protection Agency (2019) Appel et al. (2021)
National Center for Atmospheric Research (NCAR), USA	WRF-Chem	NA	Hodzic et al. (2014) Knote et al. (2014)
University of Hertfordshire, United Kingdom	WRF/CMAQ	EU	U.S. Environmental Protection Agency (2019) Appel et al. (2021)

**Table 2.** Variables from the CAMS EAC4 reanalysis provided for the generation of initial and boundary conditions.

Trace gas species	Aerosol species
O_3_ (ozone)	Sea salt aerosol at 80 % relative humidity (wet radii 0.03–0.5 μm)*
CO (carbon monoxide)	Sea salt aerosol at 80 % relative humidity (wet radii 0.5–5 μm)*
NO (nitrogen monoxide; nitric oxide)	Sea salt aerosol at 80 % relative humidity (wet radii 5–20 μm)*
NO_2_ (nitrogen dioxide)	Dust aerosol at 0 % relative humidity (dry radii 0.03–0.55 μm)
PAN (peroxyacetyl nitrate)	Dust aerosol at 0 % relative humidity (dry radii 0.55–0.9 μm)
HNO_3_ (nitric acid)	Dust aerosol at 0 % relative humidity (dry radii 0.9–20 μm)
CH_2_O (formaldehyde)	Hydrophobic organic matter aerosol at 0 % relative humidity
SO_2_ (sulfur dioxide)	Hydrophilic organic matter aerosol at 0 % relative humidity
H_2_O_2_ (hydrogen peroxide)	Hydrophobic black carbon aerosol at 0 % relative humidity
CH_3_COCH_3_ (acetone)	Hydrophilic black carbon aerosol at 0 % relative humidity
C_2_H_6_ (ethane)	Sulfate aerosol at 0 % relative humidity
PAR (paraffins)	
CH_3_OH (methanol)	
C_3_H_8_ (propane)	
C_2_H_5_OH (ethanol)	
C_2_H_4_ (ethene)	
ALD2 (aldehydes)	
OLE (olefins)	
C_5_H_8_ (isoprene)	
HCOOH (formic acid)	
CH_3_OOH (methylperoxide)	
ONIT (organic nitrates)	

* Based on guidance from ECMWF, participants were advised to transform the provided values back to dry matter by applying a reduction factor of 4.3 for the mass mixing ratios and a reduction factor of 1.99 for the radii of the sea salt bin limits.

**Table 3.** Generic land use and land cover types for AQMEII4.

Generic LULC categories for remapping
Water
Developed/urban
Barren
Evergreen needleleaf forest
Deciduous needleleaf forest
Evergreen needleleaf forest
Deciduous needleleaf forest
Mixed forest
Shrubland
Herbaceous
Planted/cultivated
Grassland
Savanna
Wetlands
Tundra
Snow and ice

**Table 4.** AQMEII4 reported dry deposition diagnostic variables for gas-phase species.

Name	AQMEII4 name	Formula

*V*_d_	VD	Deposition velocity
*r*_a_	RES-AERO	Aerodynamic resistance
*r*_c_	RES-SURF	Bulk surface resistance
*r*_s_	RES-STOM	Stomatal resistance
*r*_m_	RES-MESO	Mesophyll resistance
*r*_cut_	RES-CUT	Cuticle resistance
*E*_STOM_	ECOND-ST	Effective conductance associated with deposition to plant stomata
*E*_CUT_	ECOND-CUT	Effective conductance associated with deposition to leaf cuticles
*E*_SOIL_	ECOND-SOIL	Effective conductance associated with deposition to soil and un-vegetated surfaces
*E*_LCAN_	ECOND-LCAN	Effective conductance associated with deposition to the lower canopy
*r*_b, stom_	RES-QLST	Quasi-laminar sublayer resistance associated with stomatal pathway*
*r*_b, cut_	RES-QLSL	Quasi-laminar sublayer resistance associated with cuticular pathway*
*r*_b, soil_	RES-QLSL	Quasi-laminar sublayer resistance associated with soil pathway*
*r*_b, lcan_	RES-QLLC	Quasi-laminar sublayer resistance associated with lower canopy pathway*
*r*_dc_	RES-CONV	Resistance associated with within-canopy buoyant convection

Post-processing fields: effective conductances times net flux divided by deposition velocity		
DFLX-LCAN		Fraction of flux via lower canopy pathway
DFLX-ST		Fraction of flux via stomatal pathway
DFLX-CUT		Fraction of flux via cuticle pathway
DFLX-SOIL		Fraction of flux via soil pathway

* Equal to *r*_b_ if this is pathway-independent for the resistance framework.

**Table 5.** AQMEII4 dry deposition diagnostic variables for gas-phase species corresponding to the resistance framework of Fig. 2a.

Name	AQMEII4 name = resistance diagram variable name or formula
*r*_a_	RES-AERO = *r*_a_
*r*_c_	RES-SURF = ${\left({\left(r_{\text{leaf}1}+{\left({\left(r_{\text{stom}1}+r_{\text{m}}\right)}^{-1}+{\left(r_{\text{cut}1}\right)}^{-1}\right)}^{-1}\right)}^{-1}+{\left(r_{\text{soil1}}+r_{\text{soil2}}+r_{\text{soil3}}\right)}^{-1}\right)}^{-1}$
*r*_s_	RES-STOM = *r*_stom1_
*r*_m_	RES-MESO = *r*_m_
*r*_cut_	RES-CUT = *r*_cut1_
*E*_STOM_	ECOND-ST = $\left(\frac{{\left(r_{\text{stom}1}+r_{\text{m}}\right)}^{-1}}{{\left(r_{\text{stom}1}+r_{\text{m}}\right)}^{-1}+{\left(r_{\text{cut1}}\right)}^{-1}}\right)\;(\frac{{\left(r_{\text{leaf}1}+{\left({\left(r_{\text{stom}1}+r_{\text{m}}\right)}^{-1}+{\left(r_{\text{cut1}}\right)}^{-1}\right)}^{-1}\right)}^{-1}}{{\left(r_{\text{leafl}}+{\left({\left(r_{\text{stom}1}+r_{\text{m}}\right)}^{-1}+{\left(r_{\text{cut}1}\right)}^{-1}\right)}^{-1}\right)}^{-1}+{\left(r_{\text{soil1}}+r_{\text{soil2}}+r_{\text{soil3}}\right)}^{-1}})V_{\text{d}}$
*E*_CUT_	ECOND-CUT= $\left(\frac{{\left(r_{\text{cut1}}\right)}^{-1}}{{\left(r_{\text{stom}1}+r_{\text{m}}\right)}^{-1}+{\left(r_{\text{cut1}}\right)}^{-1}}\right)\;\left(\frac{{\left(r_{\text{leaf}1}+{\left({\left(r_{\text{stom}1}+r_{\text{m}}\right)}^{-1}+{\left(r_{\text{cut1}}\right)}^{-1}\right)}^{-1}\right)}^{-1}}{{\left(r_{\text{leafl}}+{\left({\left(r_{\text{stom}1}+r_{\text{m}}\right)}^{-1}+{\left(r_{\text{cut}1}\right)}^{-1}\right)}^{-1}\right)}^{-1}+{\left(r_{\text{soill}}+r_{\text{soil2}}+r_{\text{soil3}}\right)}^{-1}}\right)V_{\text{d}}$
*E*_SOIL_	ECOND-SOIL = $\left(\frac{{\left(r_{\text{soil}1}+r_{\text{soil2}}+r_{\text{soil3}}\right)}^{-1}}{{\left(r_{\text{leaf}1}+{\left({\left(r_{\text{stom}1}+r_{\text{m}}\right)}^{-1}+{\left(r_{\text{cut}1}\right)}^{-1}\right)}^{-1}\right)}^{-1}+{\left(r_{\text{soill}}+r_{\text{soil2}}+r_{\text{soil3}}\right)}^{-1}}\right)V_{\text{d}}$
*E*_LCAN_	ECOND-LCAN= −9
*r*_b, stom_	RES-QLST= *r*_leaf1_
*r*_b, cut_	RES-QLCT= *r*_leaf1_
*r*_b, soil_	RES-QLCL= *r*_soil2_
*r*_b, lcan_	RES-QLLC= −9
*r*_dc_	RES-CONV= −9

**Table 6.** AQMEII4 dry deposition diagnostic variables for gas-phase species corresponding to the resistance framework of Fig. 2b.

Name	AQMEII4 name = resistance diagram variable name or formula
*r*_a_	RES-AERO = *r*_a_
*r*_c_	RES-SURF = ${\left({\left(r_{\text{s}}+r_{\text{m}}\right)}^{-1}+{\left(r_{\text{lu}}\right)}^{-1}+{\left(r_{\text{soil}1}+r_{\text{soi}2}\right)}^{-1}\right)}^{-1}$
*r*_s_	RES-STOM = *r*_s_
*r*_m_	RES-MESO = *r*_m_
*r*_cut_	RES-CUT = *r*_lu_
*E*_STOM_	ECOND-ST = $\left(\frac{{\left(r_{\text{s}}+r_{\text{m}}\right)}^{-1}}{{\left(r_{\text{s}}+r_{\text{m}}\right)}^{-1}+{\left(r_{\text{lu}}\right)}^{-1}+{\left(r_{\text{soill}}+r_{\text{soil2}}\right)}^{-1}}\right)V_{\text{d}}$
*E*_CUT_	ECOND-CUT = $\left(\frac{{\left(r_{\text{lu}}\right)}^{-1}}{{\left(r_{\text{s}}+r_{\text{m}}\right)}^{-1}+{\left(r_{\text{lu}}\right)}^{-1}+{\left(r_{\text{soill}}+r_{\text{soi}2}\right)}^{-1}}\right)V_{\text{d}}$
*E*_SOIL_	ECOND-SOIL = $\left(\frac{{\left(r_{\text{soil1}}+r_{\text{soil2}}\right)}^{-1}}{{\left(r_{\text{s}}+r_{\text{m}}\right)}^{-1}+{\left(r_{\text{lu}}\right)}^{-1}+{\left(r_{\text{soil}1}+r_{\text{soil2}}\right)}^{-1}}\right)V_{\text{d}}$
*E*_LCAN_	ECOND-LCAN = −9
*r*_b, stom_	RES-QLST = *r*_b_
*r*_b, cut_	RES-QLCT = *r*_b_
*r*_b, soil_	RES-QLSL = *r*_b_
*r*_b, lcan_	RES-QLLC = −9
*r*_dc_	RES-CONV = −9

**Table 7.** Variables for bidirectional fluxes of ammonia.

Name as described here	AQMEII4 variable name	Details
*r*_sum_	RES-SUM-NH3	Net bidirectional flux ammonia resistance
*r*_a_	RES-AERO-NH3	Net aerodynamic resistance used for ammonia bidirectional fluxes
*c*_a_	CONC-NH3-AIR	Air concentration of ammonia used for bidirectional flux calculations
*c*_c_	COMP-NH3-NET	Net ammonia overall compensation point concentration
*c*_g_	COMP-NH3-GND	Net ammonia compensation point concentration with respect to ground
*c*_s_	COMP-NH3-STO	Net ammonia compensation point concentration with respect to stomata

**Figure B1.**
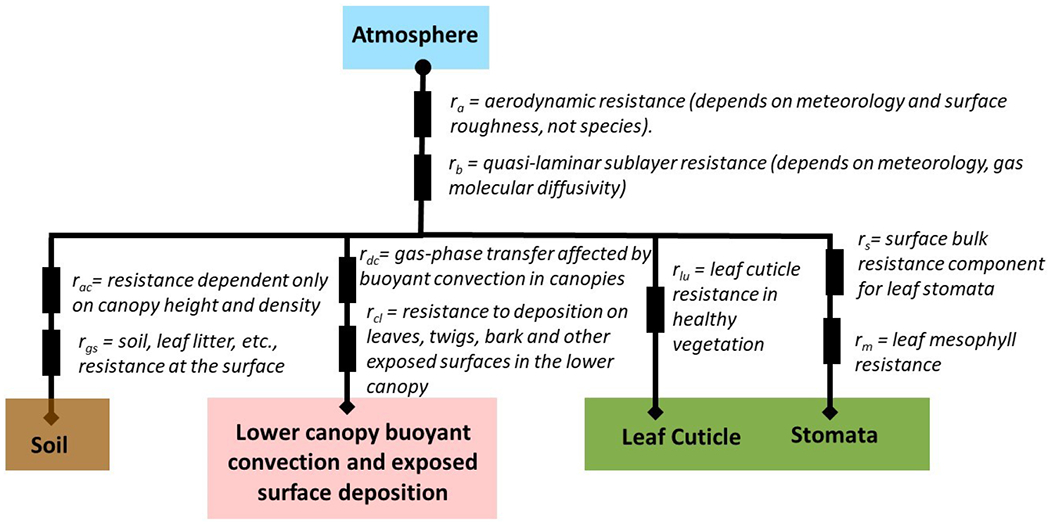
Resistance diagram for the ECCC GEM-MACH model (default Robichaud scheme).

**Figure B5.**
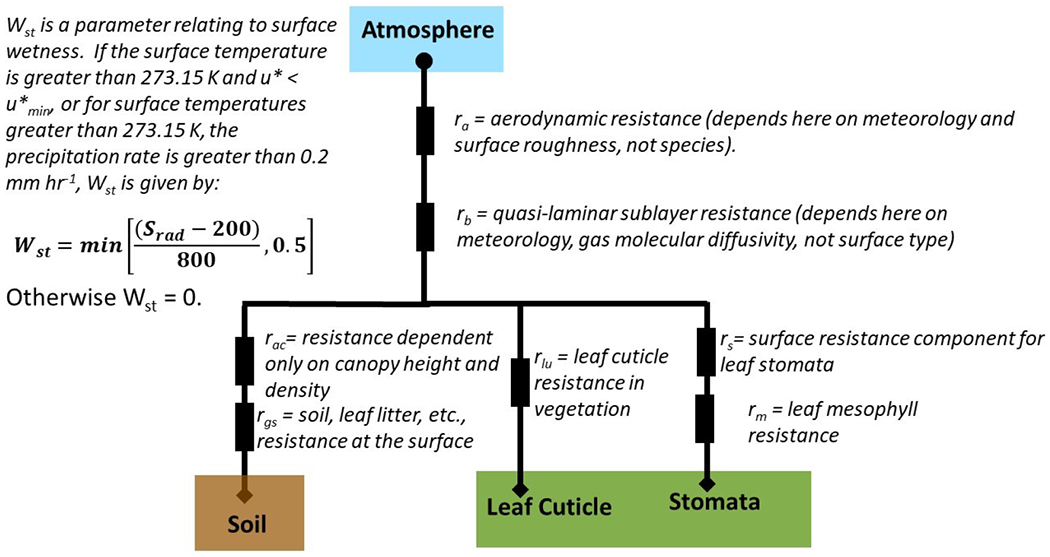
Resistance diagram for the ECCC GEM-MACH model (Zhang scheme).

**Figure B6.**
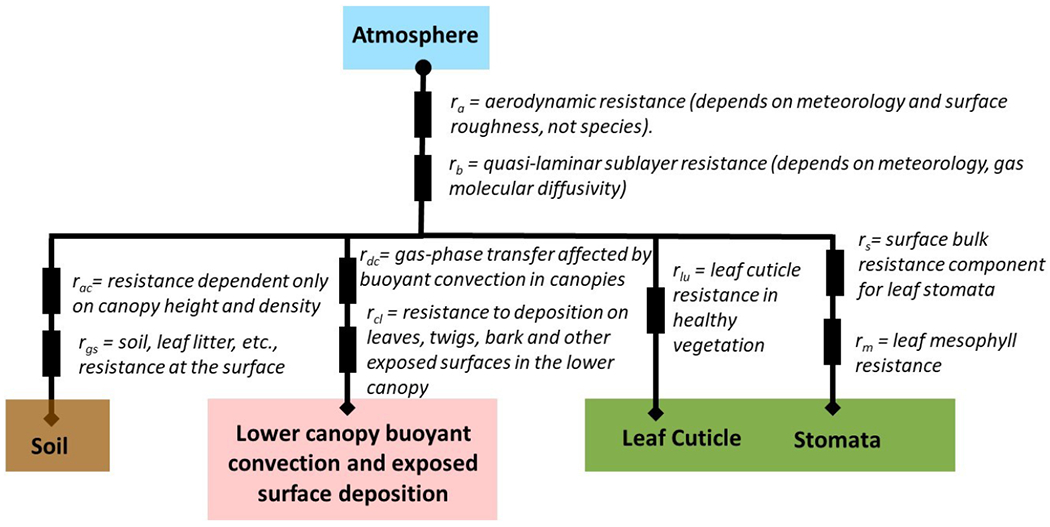
Resistance diagram for the gaseous dry deposition scheme implemented in WRF-Chem.

**Figure B7.**
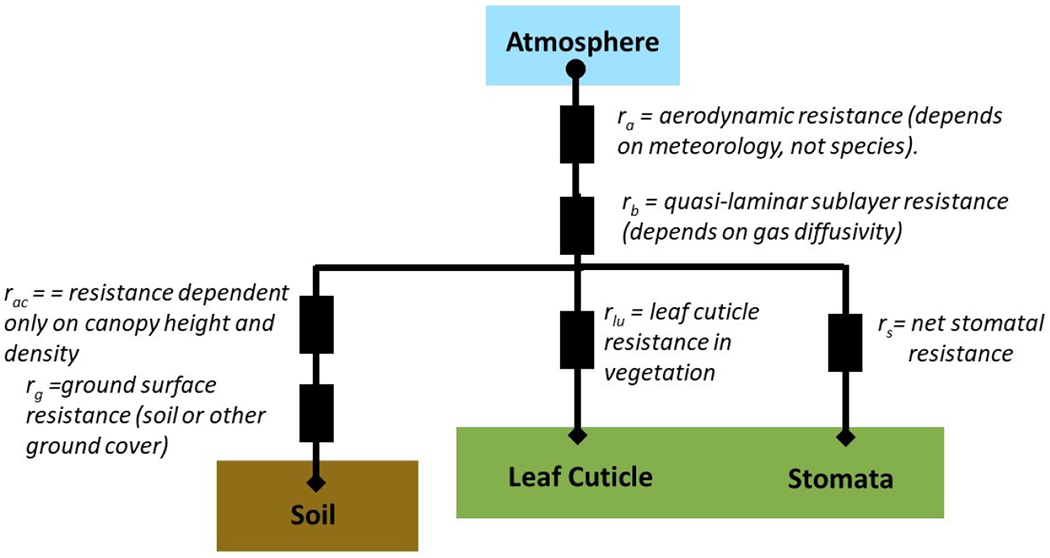
Resistance diagram for the dry deposition scheme implemented in COSMO-MUSCAT.

## Data Availability

No datasets were used in this article.

## Footnotes

1 Note that the depositing gases in each pathway are influenced by *r*_a_ and *r*_b_ prior to encountering the different resistances that make up *r*_c_, and this is why *v*_d_, which includes the influence of *r*_a_ and *r*_b_, is scaled by the fraction of the inverse of rc occurring through a given pathway. Some models include surface-specific quasi-laminar sublayer resistances; when this is the case, these terms appear in the pathway-specific fractions of the total uptake terms.

## Appendix A: Standard output requested from all participating models

**Table A1.** AQMEII4 – meteorology (grid).

Variable	Description and units
PRECIP	Sum of all surface precipitation, cm
PRESS	Surface pressure, hPa
MIXRAT	Water vapour mixing ratio at 2 m, g kg^−1^
RH	Relative humidity at 2 m, %
TD	Dew point temperature at 2 m, K
TEMP	Air temperature at 2 m, K
WS	Horizontal wind speed at 10 m, m s^−1^
WD	Horizontal wind direction at 10 m, °
W	Vertical wind speed at 10 m, m s^−1^
SWGU	Upward shortwave radiation at the ground, W m^−2^
SWGD	Downward shortwave radiation at the ground, W m^−2^
SWTU	Upward shortwave radiation at atmosphere top, W m^−2^
SWTD	Downward shortwave radiation at atmosphere top, W m^−2^
PBL	Planetary boundary layer height, m
PAR	Photosynthetically active radiation at the ground, W m^−2^
AOD470	Aerosol optical depth at 470 nm
AOD555	Aerosol optical depth at 555 nm
AOD675	Aerosol optical depth at 675 nm
H2O	Water vapour column, cm^3^ cm^−2^
USTAR	Friction velocity, ms^−1^
MOL	Monin–Obukhov length, m
RHO	Air density of lowest model layer
TEMP10	Air temperature at 10 m, K
TSOIL	Uppermost soil layer temperature, K
SNOWC	Fractional coverage of snow in grid cell, 0–1
WETCAN	Canopy wetness, 0.0 if dry and 1.0 if wet
SOILMOI	Uppermost soil layer moisture, m^3^ m^−3^
Z0	Surface roughness length, m
ALB	Albedo, fraction
Z	Terrain height above sea level, m
FWET	Wet surface, unitless fraction
LAI-T	Total leaf area index, m^2^ m^−2^

**Table A2.** AQMEII4 – gas and particle concentrations and emissions (grid).

Variable	Description and units
SO2	Concentration of SO_2_ at ground, μg m^−3^
NO2	Concentration of NO_2_ at ground, μg m^−3^
NO	Concentration of NO at ground, μg m^−3^
NOx	Concentration of NO_*x*_ at ground, μg m^−3^
NOy	Concentration of NO_*y*_ at ground, μg m^−3^
HNO3	Concentration of HNO_3_ at ground, μg m^−3^
NH3	Concentration of NH_3_ at ground, μg m^−3^
PAN	Concentration of PAN at ground, μg m^−3^
HNO4	Concentration of HNO_4_ at ground, μg m^−3^
N2O5	Concentration of N_2_O_5_ at ground, μg m^−3^
HONO	Concentration of HONO at ground, μg m^−3^
ONIT	Concentration of gaseous organic nitrates at ground, μg m^−3^
O3	Concentration of O_3_ at ground, μg m^−3^
H2O2	Concentration of H_2_O_2_ at ground, μg m^−3^
HCHO	Concentration of formaldehyde at ground, μg m^−3^
CO	Concentration of CO at ground, μg m^−3^
ETHE	Concentration of ethene at ground, μg m^−3^
C5H8	Concentration of isoprene at ground, μg m^−3^
C10H16	Concentration of monoterpenes at ground, μg m^−3^
PM2_5_SU	Concentration of PM_2.5_ sulfate at ground, μg m^−3^
PM2_5_AM	Concentration of PM_2.5_ ammonium at ground, μg m^−3^
PM2_5_NI	Concentration of PM_2.5_ nitrate at ground, μg m^−3^
PM2_5_POA	Concentration of PM_2.5_ primary organic aerosol at ground, μg m^−3^
PM2_5_SOA	Concentration of PM_2.5_ secondary organic aerosol at ground, μg m^−3^
PM2_5_OC	Concentration of PM_2.5_ organic carbon at ground, μg m^−3^
PM2_5_EC	Concentration of PM_2.5_ elemental carbon (black carbon) at ground, μg m^−3^
PM2_5_SS	Concentration of PM_2.5_ sea salt at ground, μg m^−3^
PM2_5_CA	Concentration of PM_2.5_ calcium at ground, μg m^−3^
PM2_5_MG	Concentration of PM_2.5_ magnesium at ground, μg m^−3^
PM2_5_NSNA	Concentration of PM_2.5_ non-sea-salt sodium at ground, μg m^−3^
PM2_5_PK	Concentration of PM_2.5_ potassium at ground, μg m^−3^
PM2_5_FE	Concentration of PM_2.5_ iron at ground, μg m^−3^
PM2_5_MN	Concentration of PM_2.5_ manganese at ground, μg m^−3^
PM2_5_OTH	Concentration of PM_2.5_ other (all not speciated) at ground, μg m^−3^
PM10_SU	Concentration of PM_10_ sulfate at ground, μg m^−3^
PM10_AM	Concentration of PM_10_ ammonium at ground, μg m^−3^
PM10_NI	Concentration of PM_10_ nitrate at ground, μg m^−3^
PM10_POA	Concentration of PM_10_ primary organic aerosol at ground, μg m^−3^
PM10_SOA	Concentration of PM_10_ secondary organic aerosol at ground, μg m^−3^
PM10_OC	Concentration of PM_10_ organic carbon (at ground, μg m^−3^
PM10_EC	Concentration of PM_10_ elemental carbon (black carbon) at ground, μg m^−3^
PM10_SS	Concentration of PM_10_ sea salt at ground, μg m^−3^
PM10_CA	Concentration of PM_10_ calcium at ground, μg m^−3^
PM10_MG	Concentration of PM_10_ magnesium at ground, μg m^−3^
PM10_NSNA	Concentration of PM_10_ non-sea-salt sodium at ground, μg m^−3^
PM10_PK	Concentration of PM_10_ potassium at ground, μg m^−3^
PM10_FE	Concentration of PM_10_ iron at ground, μg m^−3^
PM10_MN	Concentration of PM_10_ manganese at ground, μg m^−3^
PM10_OTH	Concentration of PM_10_ other (all not speciated) at ground, μg m^−3^
PMTOT_SU	Concentration of PMTOT sulfate at ground, μg m^−3^
PMTOT_AM	Concentration of PMTOT ammonium at ground, μg m^−3^
PMTOT_NI	Concentration of PMTOT nitrate at ground, μg m^−3^
PMTOT_POA	Concentration of PMTOT primary organic aerosol at ground, μg m^−3^
PMTOT_SOA	Concentration of PMTOT secondary organic aerosol at ground, μg m^−3^
PMTOT_OC	Concentration of PMTOT organic carbon at ground, μg m^−3^
PMTOT_EC	Concentration of PMTOT elemental carbon (black carbon) at ground, μg m^−3^
PMTOT_SS	Concentration of PMTOT sea salt at ground, μg m^−3^
PMTOT_CA	Concentration of PMTOT calcium at ground, μg m^−3^
PMTOT_MG	Concentration of PMTOT magnesium at ground, μg m^−3^
PMTOT_NSNA	Concentration of PMTOT non-sea-salt sodium at ground, μg m^−3^
PMTOT_PK	Concentration of PMTOT potassium at ground, μg m^−3^
PMTOT_FE	Concentration of PMTOT iron at ground, μg m^−3^
PMTOT_MN	Concentration of PMTOT manganese at ground, μg m^−3^
PMTOT_OTH	Concentration of PMTOT other (all not speciated) at ground, μg m^−3^
PM2_5	Concentration of PM_2.5_ at ground, μg m^−3^
PM2_5N	Number concentration of PM_2.5_ at ground, cm^−3^
PM10	Concentration of PM_10_ at ground, μg m^−3^
PM10N	Number concentration of PM_10_ at ground, cm^−3^
PMTOT	Concentration of total PM at ground, μg m^−3^
PMTOTN	Number concentration of total PM at ground, cm^−3^
JNO2	Photolysis rate of NO_2_ at ground, 1E-3 s^−1^
E_SO2	Accumulated emission of SO_2_, kg km^−2^
E_ANOX	Accumulated emission of anthropogenic NO + NO_2_ as NO_2_, kg km^−2^
E_NH3	Accumulated emission of NH_3_, kg km^−2^
E_CO	Accumulated emission of CO, kg km^−2^
E_PM2_5	Accumulated emission of primary PM_2.5_, kg km^−2^
E_PM10	Accumulated emission of primary PM_10_, kg km^−2^
E_ETHE	Accumulated emission of ethene, kg-C km^−2^
E_TOLU	Accumulated emission of toluene, kg-C km^−2^
E_HCHO	Accumulated emission of formaldehyde, kg-C km^−2^
E_C5H8	Accumulated emission of isoprene, kg-C km^−2^
E_MNTP	Accumulated emission of monoterpenes, kg-C km^−2^
E_SQTP	Accumulated emission of sesquiterpenes, kg-C km^−2^
E_OVOC	Accumulated emission of other VOCs not in above groups, kg-C km^−2^
E_SNOX	Accumulated emission of soil NO+NO_2_ as NO_2_, kg km^−2^
E_SS	Accumulated emission of sea salt (all particle sizes), kg km^−2^
E_WBDUST	Accumulated emission of wind-blown dust (all particle sizes), kg km^−2^
PM2_5_WAT	Concentration of PM_2.5_ water at ground (if calculated), μg m^−3^
PM10_WAT	Concentration of PM_10_ water at ground (if calculated), μg m^−3^
PMTOT_WAT	Concentration of PMTOT water at ground (if calculated), μg m^−3^

**Table A3.** AQMEII4 – deposition fluxes (grid).

WFLUX-HSO3−	Wet deposition flux of ${\mathrm{HSO}}_{3}^{-}$ ion, eq ha^−1^
WFLUX-SO4=	Wet deposition flux of ${\mathrm{SO}}_{4}^{=}$ ion, eq ha^−1^
WFLUX-NO3−	Wet deposition flux of ${\mathrm{NO}}_{3}^{-}$ ion, eq ha^−1^
WFLUX-NH4+	Wet deposition flux of ${\mathrm{NH}}_{4}^{+}$ ion, eq ha^−1^
WFLUX-BCT1	Wet deposition flux of base cations, eq ha^−1^
WFLUX-TOC	Wet deposition flux of total organic carbon, g ha^−1^
PRECIP	Surface precipitation, cm
DFLUX-SO2	Dry deposition flux of sulfur dioxide gas, eq ha^−1^
DFLUX-NO2	Dry deposition flux of nitrogen dioxide gas, eq ha^−1^
DFLUX-NO	Dry deposition flux of nitrogen monoxide gas, eq ha^−1^
DFLUX-HNO3	Dry deposition flux of nitric acid gas, eq ha^−1^
DFLUX-NH3	Net flux of ammonia gas (negative if upwards), eq ha^−1^
DFLUX-PAN	Dry deposition flux of peroxyacetyl nitrate gas, eq ha^−1^
DFLUX-HNO4	Dry deposition flux of peroxynitric acid gas, eq ha^−1^
DFLUX-N2O5	Dry deposition flux of dinitrogen pentoxide gas, eq ha^−1^
DFLUX-ONIT	Dry deposition flux of gaseous organic nitrate, eq ha^−1^
DFLUX-O3	Dry deposition flux of ozone gas, g ha^−1^
DFLUX-H2O2	Dry deposition flux of hydrogen peroxide gas, g ha^−1^
DFLUX-HCHO	Dry deposition flux of formaldehyde gas, g ha^−1^
DFLUX-P-SO4	Dry deposition flux of total particle sulfate, eq ha^−1^
DFLUX-P-NO3	Dry deposition flux of total particle nitrate, eq ha^−1^
DFLUX-P-NH4	Dry deposition flux of total particle ammonium, eq ha^−1^
DFLUX-P-TC	Dry deposition flux of total particle organic carbon, g ha^−1^
DFLUX-P-EC	Dry deposition flux of total black carbon, g ha^−1^
DFLUX-P-BCT1	Dry deposition flux of total particulate base cations, eq ha^−1^
WFLUX-HSO3−	Wet deposition flux of ${\mathrm{HSO}}_{3}^{-}$ ion, eq ha^−1^
DFLUX-P-BCT2	Flux of base cat. removed as non-transportable fraction during emissions processing (if available), eq ha^−1^
DFLUX-P-SS	Dry deposition flux of total sea salt aerosol, mol ha^−1^
DFLUX-P-CM	Dry deposition flux of total crustal material (all particulate components not speciated above), g ha^−1^
DFLUX-PM2_5	Dry deposition flux of PM_2.5_, g ha^−1^
DFLUX-HONO	Dry deposition flux of HONO, eq ha^−1^
RES-AERO	Aerodynamic resistance, s cm^−1^

## Appendix B: Resistance diagrams and calculation of AQMEII4 reported dry deposition diagnostic variables for dry deposition schemes implemented in participating models

### B1 Example 1: GEM-MACH model, default Robichaud scheme

These are the calculations for the Environment and Climate Change Canada model GEM-MACH (Global Environmental Multiscale- Modelling Air-quality and CHemistry). The resistance diagram for this model is shown in Fig. B1. The deposition algorithm closely follows Wesely’s original, hence the similarities to Fig. 2. The scheme includes further modifications incorporating parameterizations from Jarvis (1976), Val Martin et al. (2014), and other authors; details and references for this scheme may be found in Makar et al. (2018), Supplement). In GEM-MACH, snow, when present, is treated as a separate land use type.

The main difference between the resistances in Wesely (1989) and the GEM-MACH resistances (aside from formulation details) is the addition of a surface wetness term, (1 – *W*_st_), intended to account for the influence of wet surfaces on dry deposition.

**Table B1.** Example 1: AQMEII4-reported gaseous deposition variables corresponding to the GEM-MACH Robichaud resistance model of Fig. B1.

Name as described here	AQMEII4 name = resistance diagram variable name or formula
*r*_a_	RES-AERO = *r*_a_
*r*_c_	RES-SURF = $\frac{1}{\frac{\left(1-W_{\text{St}}\right)}{\left(r_{\text{S}}+r_{\text{m}}\right)}+\frac{1}{r_{\text{lu}}}+\frac{1}{r_{\text{dc}}+r_{\text{cl}}}+\frac{1}{r_{\text{ac}}+r_{\text{gs}}}}$
*r*_s_	RES-STOM = *r*_s_
*r*_m_	RES-MESO = *r*_m_	
*r*_cut_	RES-CUT = *r*_lu_
*E*_STOM_	ECOND-ST= $\left(\frac{\frac{\left(1-W_{\text{st}}\right)}{\left(r_{\text{s}}+r_{\text{m}}\right)}}{\frac{\left(1-W_{\text{st}}\right)}{\left(r_{\text{s}}+r_{\text{m}}\right)}+\frac{1}{r_{\text{lu}}}+\frac{1}{r_{\text{dc}}+r_{\text{cl}}}+\frac{1}{r_{\text{ac}}+r_{\text{gs}}}}\right)V_{\text{d}}$
*E*_CUT_	ECOND-CUT = $\left(\frac{\frac{1}{r_{\text{lu}}}}{\frac{\left(1-W_{\text{st}}\right)}{\left(r_{\text{s}}+r_{\text{m}}\right)}+\frac{1}{r_{\text{lu}}}+\frac{1}{r_{\text{dc}}+r_{\text{cl}}}+\frac{1}{r_{\text{ac}}+r_{\text{gs}}}}\right)V_{\text{d}}$	
*E*_SOIL_	ECOND-SOIL= $\left(\frac{\frac{1}{r_{\text{ac}}+r_{\text{gs}}}}{\frac{\left(1-W_{\text{st}}\right)}{\left(r_{\text{s}}+r_{\text{m}}\right)}+\frac{1}{r_{\text{lu}}}+\frac{1}{r_{\text{dc}}+r_{\text{cl}}}+\frac{1}{r_{\text{ac}}+r_{\text{gs}}}}\right)V_{\text{d}}$
*E*_LCAN_	ECOND-LCAN = $\left(\frac{\frac{1}{r_{\text{dc}}+r_{\text{cl}}}}{\frac{\left(1-W_{\text{st}}\right)}{\left(r_{\text{s}}+r_{\text{m}}\right)}+\frac{1}{r_{\text{lu}}}+\frac{1}{r_{\text{dc}}+r_{\text{cl}}}+\frac{1}{r_{\text{ac}}+r_{\text{gs}}}}\right)V_{\text{d}}$
*r*_b, stom_	RES-QLST = *r*_b_
*r*_b, cut_	RES-QLCT = *r*_b_
*r*_b, soil_	RES-QLSL = *r*_b_
*r*_b, lcan_	RES-QLLC = *r*_b_
*r*_dc_	RES-CONV= *r*_dc_

### B2 Example 2: CMAQ M3DRY

The second specific air quality model example is the M3DRY algorithm implemented in the U.S. EPA’s Community Multiscale Air Quality (CMAQ) model, one of two available dry deposition options in that model. In this particular case, separate branches occur for the vegetated versus non-vegetated fraction within each model grid cell, and further branching resistance pathways take into account the fraction of the grid cell which is wet versus dry and snow-covered versus non-snow-covered. In-canopy convective effects are only calculated for the vegetated fraction.

**Table B2.** AQMEII4-reported gaseous deposition variables corresponding to the CMAQ M3Dry resistance model of Fig. B2.

Name as described here	AQMEII4 name = resistance diagram variable name or formula
*r*_a_	RES-AERO = *r*_a_
*r*_c_	RES-SURF = $\frac{1}{\left\{F_{\text{veg}}(\frac{1}{r_{\text{s}}+r_{\text{m}}}+\frac{\left(1-F_{\text{wet}}\right)\text{LAI}}{r_{\text{cut, dry}}}+\frac{F_{\text{wet}}\cdot \text{LAI}}{r_{\text{cut, wet}}}+\frac{1}{r_{\text{dc}}+\frac{1}{\left(1-\text{if snow}\right)\left(\frac{\left(1-F_{\text{wet}}\right)}{r_{\text{soil, dry}}}+\frac{F_{\text{wet}}}{r_{\text{soil, wet}}}\right)+\left(\text{if snow}\right)\left(\frac{\left(1-x_{\text{m}}\right)}{r_{\text{snow, dry}}}+\frac{x_{\text{m}}}{r_{\text{sndiff}}+r_{\text{snow, wet}}}\right)}}+\left(1-F_{\text{veg}}\right)\left(\left(1-\text{if snow}\right)\left(\frac{\left(1-F_{\text{wet}}\right)}{r_{\text{soil, dry}}}+\frac{F_{\text{wet}}}{r_{\text{soil, wet}}}\right)+\left(\text{if snow}\right)\left(\frac{\left(1-x_{\text{m}}\right)}{r_{\text{snow, dry}}}+\frac{x_{\text{m}}}{r_{\text{sndiff}}+r_{\text{snow, wet}}}\right)\right)\right\}}$
*r*_s_	RES-STOM = *r*_s_
*r*_m_	RES-MESO = r_m_
*r*_cut_	RES-CUT= $\frac{1}{\frac{\left(1-F_{\text{wet}}\right)\mathrm{LAI}}{r_{\text{cut, dry}}}+\frac{F_{\text{wet}}\cdot \mathrm{LAI}}{r_{\text{cut, wet}}}}$
*E*_STOM_	ECOND-ST = $\left[\frac{\left(F_{\text{veg}}\right)}{\left(r_{\text{s}}+r_{\text{m}}\right)}\right]$ (RES-SURF) *V*_d_
*E*_CUT_	ECOND-CUT = (RES-CUT)^−1^ (RES-SURF) *V*_d_
*E*_SOIL_	ECOND-SOIL
	$=\left[\left(1-F_{\text{veg}}\right)\left((1-\text{if snow})\left(\frac{\left(1-F_{\text{wet}}\right)}{r_{\text{soil, dry}}}+\frac{F_{\text{wet}}}{r_{\text{soil, wet}}}\right)+(\text{if snow})\left(\frac{\left(1-x_{\text{m}}\right)}{r_{\text{snow, dry}}}+\frac{x_{\text{m}}}{r_{\text{sndiff}}+r_{\text{snow, wet}}}\right)\right)\right]\text{(RES-SURF)}V_{\text{d}}$
*E*_LCAN_	ECOND-LCAN = $=\left[\frac{F_{\text{veg}}}{r_{\text{dc}}+\frac{1}{\left(1-\text{if snow}\right)\left(\frac{\left(1-F_{\text{wet}}\right)}{r_{\text{soil, dry}}}+\frac{F_{\text{wet}}}{r_{\text{soil, wet}}}\right)+\left(\text{if snow}\right)\left(\frac{\left(1-x_{\text{m}}\right)}{r_{\text{snow, dry}}}+\frac{x_{\text{m}}}{r_{\text{sndiff}}+r_{\text{snow, wet}}}\right)}}\right]$ (RES-SURF) *V*_d_
*r*_b, stom_	RES-QLST = *r*_b_
*r*_b, cut_	RES-QLCT = *r*_b_
*r*_b, soil_	RES-QLSL = *r*_b_
*r*_b, lcan_	RES-QLLC = *r*_b_
*r*_dc_	RES-CONV = *r*_dc_

Note that the vegetated fraction and leaf area index used in the above equations for CMAQ with the M3DRY deposition option are for specific LULC types: the quantities in Table B2 will be reported for each of the 16 generic LULC categories for AQMEII4. Note that the lower canopy pathway has been identified as such due to the presence of the *r*_dc_ term; i.e. this points to its similarity with Wesely’s original lower canopy pathway.

### B3 Example 3: CMAQ STAGE

The third specific air quality model example is the algorithm used by the U.S. EPA’s Community Multiscale Air Quality (CMAQ) model with the Surface Tiled Aerosol and Gaseous Exchange (STAGE) deposition option. In this particular case, separate branches occur for the vegetated versus non-vegetated fraction for each LULC type within each model grid cell, and further branching resistance pathways take into account the fraction of the grid cell which is wet versus dry and snow-covered versus non-snow-covered. Incanopy convective effects are only calculated for in the vegetated fraction.

**Table B3.** AQMEII4-reported gaseous deposition variables corresponding to the CMAQ STAGE resistance model of Fig. B3.

Name as described here	AQMEII4 name = resistance diagram variable name or formula
*r*_a_	RES-AERO = *r*_a_
*r*_c_	RES-SURF = $\frac{1}{\left\{\frac{F_{\text{veg}}}{r_{\text{can, qlsb}}+\frac{1}{\frac{1}{r_{\text{s}}+r_{\text{m}}}+\frac{F_{\text{dry}}\;\text{LAI}}{r_{\text{cut, dry}}}+\frac{F_{\text{wet}}\;\text{LAI}}{r_{\text{cut, wet}}}}+\frac{1}{r_{\text{dc}}+r_{\text{gnd, qlsb}}+\frac{1}{F_{\text{snow}}\left(\frac{F_{\text{frozen}}}{r_{\text{sndiff}}+r_{\text{snow, wet}}}+\frac{F_{\text{melting}}}{r_{\text{snow, dry}}}\right)+F_{\text{snowfree}}\left(\frac{F_{\text{dry}}}{r_{\text{soil, dry}}}+\frac{F_{\text{wet}}}{r_{\text{soil, wet}}}\right)}}+\frac{F_{\text{no-veg}}}{r_{\text{gnd, qlsb}}+\frac{1}{F_{\text{snow}}\left(\frac{F_{\text{frozen}}}{r_{\text{sndiff}}+r_{\text{snow, wet}}}+\frac{F_{\text{melting}}}{r_{\text{snow, dry}}}\right)+F_{\text{snowfree}}\left(\frac{F_{\text{dry}}}{r_{\text{soil, dry}}}+\frac{F_{\text{wet}}}{r_{\text{soil, wet}}}\right)}}}\right\}}$
*r*_s_	RES-STOM = *r*_s_
*r*_m_	RES-MESO = *r*_m_
*r*_cut_	RES-CUT = *r*_cut_
*E*_STOM_	ECOND-ST = $\left[\;[\frac{\left(F_{\text{veg}}\right)}{\left(r_{\text{s}}+r_{\text{m}}\right)\left(r_{\text{can, qlsb}}\left\{\frac{1}{r_{\text{s}}+r_{\text{m}}}+\frac{F_{\text{dry}}\;\text{LAI}}{r_{\text{cut, dry}}}+\frac{F_{\text{wet}}\;\text{LAI}}{r_{\text{cut, wet}}}\right\}+1\right)}]\;\right]$ (RES-SURF) *V*_d_ Or ECOND-ST = $\left[\frac{\frac{1}{\left(r_{\text{s}}+r_{\text{m}}\right)}}{\frac{1}{r_{\text{s}}+r_{\text{m}}}+\frac{F_{\text{dry}}\;\text{LAI}}{r_{\text{cut, dry}}}+\frac{F_{\text{wet}}\;\text{LAI}}{r_{\text{cut, wet}}}}\right]\;\left[\frac{\left(F_{\text{veg}}\right)}{r_{\text{can, qlsb}}+\frac{1}{\frac{1}{r_{\text{s}}+r_{\text{m}}}+\frac{F_{\text{dry}}\;\text{LAI}}{r_{\text{cut, dry}}}+\frac{F_{\text{wet}}\;\text{LAI}}{r_{\text{cut, wet}}}}}\right]$ (RES-SURF) *V*_d_
*E*_CUT_	ECOND-CUT = $\left[\frac{F_{\text{veg}}}{\frac{1}{\frac{F_{\text{dry}}\;\text{LAI}}{r_{\text{cut, dry}}}+\frac{F_{\text{wet}}\;\text{LAI}}{r_{\text{cut, wet}}}}\left(r_{\text{can, qlsb}}\left\{\frac{1}{r_{\text{s}}+r_{\text{m}}}+\frac{F_{\text{dry}}\;\text{LAI}}{r_{\text{cut, dry}}}+\frac{F_{\text{wet}}\;\text{LAI}}{r_{\text{cut, wet}}}\right\}+1\right)}\right]$ (RES-SURF) *V*_d_
*E*_SOIL_	ECOND-SOIL = $\left[\frac{F_{\text{no-veg}}}{r_{\text{gnd, qlsb}}+\frac{1}{F_{\text{snow}}\left(\frac{F_{\text{frozen}}}{r_{\text{sndiff}}+r_{\text{snow, wet}}}+\frac{F_{\text{melting}}}{r_{\text{snow, dry}}}\right)+F_{\text{snowfree}}\left(\frac{F_{\text{dry}}}{r_{\text{soil, dry}}}+\frac{F_{\text{wet}}}{r_{\text{soil, wet}}}\right)}}\right]$ (RES-SURF) *V*_d_
*E*_LCAN_	ECOND-LCAN = $\left[\frac{F_{\text{veg}}}{r_{\text{dc}}+r_{\text{gnd, qlsb}}+\frac{1}{F_{\text{snow}}\left(\frac{F_{\text{frozen}}}{r_{\text{sndiff}}+r_{\text{snow, wet}}}+\frac{F_{\text{melting}}}{r_{\text{snow, dry}}}\right)+F_{\text{snowfree}}\left(\frac{F_{\text{dry}}}{r_{\text{soil, dry}}}+\frac{F_{\text{wet}}}{r_{\text{soil, wet}}}\right)}}\right]$ (RES-SURF) *V*_d_
*r*_b, stom_	RES-QLST = *r*_can, qlsb_
*r*_b, cut_	RES-QLCT = *r*_can, qlsb_
*r*_b, soil_	RES-QLSL = *r*_gnd, qlsb_
*r*_b, lcan_	RES-QLLC = *r*_gnd, qlsb_
*r*_dc_	RES-CONV = *r*_dc_

*F*_Veg_ + *F*_no-veg_ = 1; vegetation coverage fractions.

*F*_snow_ + *F*_snowfree_ = 1; snow coverage fraction.

*F*_wet_ + *F*_dry_ = 1; surface wetness fractions.

*F**frozen* + *F*_melting_ = 1; snowmelt fractions.

Note that the vegetated fraction and leaf area index used in the above equations for CMAQ with the STAGE deposition option are for specific LULC types: the quantities in Table B3 will be reported for each of the 16 generic LULC categories for AQMEII4. Note that the lower canopy pathway has been identified as such due to the presence of the *r*_dc_ term; i.e. this points to its similarity with Wesely’s original lower canopy pathway.

### B4 Example 4. LOTOS EUROS

**Table B4.** AQMEII4-reported gaseous deposition variables corresponding to the LOTOS-EUROS resistance model of Fig. B4.

Name as described here	AQMEII4 name = resistance diagram variable name or formula
*r*_a_	RES-AERO = $\frac{\text{ln}\left(\frac{z_{\text{r}}}{z_{0}}\right)+4.7\left(\frac{z_{\text{r}}-z_{0}}{L}\right)}{K\cdot u*}$ for stable conditions, *κ*: von Kármán constant (here 0.35); *L*: Monin–Obukhov length; *z*_r_: reference height; *z*_0_: height of surface roughness
*r*_b_	RES-QLST = RES-QLSL = RES-QLLC = RES-QLLC = 1.3 · 150 · $\sqrt{\frac{L_{\text{d}}}{V\left(h\right)}}$, *L*_d_: cross-wind lead dimension, *V*(*h*): wind speed at canopy top *h*, factor 1.3 accounts for differences in diffusivity between heat and ozone
*r*_c_	RES-SURF = ${\left(\frac{1}{r_{w}}+\frac{1}{r_{\text{mc}}+r_{\text{soil}}}+\frac{1}{r_{\text{s}}}\right)}^{-1}$ for NO_2_, NH_3_, SO_2_, O_3_; for wet conditions, RES-SURF = 10. For HNO_3_, N_2_O_5_, NO_3_, H_2_O_2_, RES-SURF = 50 (2000 for wet conditions) For snow conditions: RES-SURF = 500 to 70. For other conditions and for NO, CO, RES-SURF= 9999.
*r_w_*	RES-CUT = 10 for wet conditions RES-CUT = 2000 for NO_2_ RES-CUT = 2500 for O_3_ RES-CUT = 25000 · *e*^(−0.0693⋅RH)^ for SO_2_ if RH < 81.3 RES-CUT = 5.8 × 10^11^ · *e*^(−0.278⋅RH)^ for SO_2_ if RH > 81.3 RES-CUT = SAI · *a* · $e^{\frac{\left(100-\text{RH}\right)}{\beta }}$ for NH_3_ SAI: surface area index; *a* = 2 s/m; *β* = 12; RH: relative humidity (%)
*r*_inc_	RES-LCAN = $\frac{b\cdot h\cdot \text{SAI}}{u^{*}}$, *b*: empirical constant (14 m^−1^); *h*: height of vegetation (m); SAI: surface area index; *u**: friction velocity (m s^−1^)
*r*_soil_	Parameterized, frozen soil, wet soil, dry soil RES-SOIL (FROZEN) = 1000 s m^−1^ for NH_3_; 2000 s m^−1^ for O_3_, NO_2_; 500 s m^−1^ for SO_2_ RES-SOIL (WET) = 10 s m^−1^ for NH_3_, SO_2_; 2000 s m^−1^ for O_3_, NO_2_ RES-SOIL (DRY) (land use dependent) 200–2000 s m^−1^ for O_3_; 10–100 s m^−1^ for NH_3_; 10–1000 s m^−1^ for SO_2_; 1000–2000 s m^−1^ for NO_2_
*r*_s_	RES-STOM = $\frac{1}{E_{\text{stom}}}$
*E*_STOM_	ECOND-ST = EMax_stom_ · *F*_light_ · *F*_phen_ · *F*_temp_ · *F*_vpd_ · *F*_swp_ · *C*_diff_ EMax: maximum stomatal conductance (derived for ozone, land use dependent) *F*_light_, *F*_phen_, *F*_temp_, *F*_vpd_, *F*_swp_: factors [0–1] for conductance dependency of light, phenology, temperature, vapour pressure, and soil water *C*_diff_: diffusion coefficient for species with respect to ozone Mesophyll conductance part incorporated in stomatal conductance
*C*_comp_	Use of compensation point to derive bidirectional flux for NH_3_ following Wichink Kruit et al. (2012), modelling the distribution of ammonia across Europe including bidirectional surface–atmosphere exchange: https://doi.org/10.5194/bg-9-5261-2012

### B5 Example 5: GEM-MACH model, Zhang scheme

These are the calculations for the Environment and Climate Change Canada model GEM-MACH (Global Environmental Multiscale- Modelling Air-quality and CHemistry), using the scheme of Zhang et al. (2002, 2003, 2010). The resistance diagram for this model is shown in Fig. B5.

The main difference in the overall construction of the deposition scheme relative to the default Robichaud scheme (aside from the details of how the different terms are calculated) is in the absence of the lower canopy buoyant convection and exposed surface deposition branch of Wesely’s original model. The details of the parameterizations for the terms in the equations also differ from the Robichaud scheme.

**Table B5.** AQMEII4-reported gaseous deposition variables corresponding to the GEM-MACH Zhang resistance model of Fig. B5.

Name as described here	AQMEII4 name = resistance diagram variable name or formula
*r*_a_	RES-AERO = *r*_a_
*r*_c_	RES-SURF = $\frac{1}{\frac{\left(1-W_{\text{st}}\right)}{\left(r_{\text{s}}+r_{\text{m}}\right)}+\frac{1}{r_{\text{lu}}}+\frac{1}{r_{\text{ac}}+r_{\text{gs}}}}$
*r*_s_	RES-STOM = *r*_s_
*r*_m_	RES-MESO = *r*_m_
*r*_cut_	RES-CUT = *r*_lu_
*E*_STOM_	ECOND-ST= $\left(\frac{\frac{\left(1-W_{\text{st}}\right)}{\left(r_{\text{s}}+r_{\text{m}}\right)}}{\frac{\left(1-W_{\text{st}}\right)}{\left(r_{\text{s}}+r_{\text{m}}\right)}+\frac{1}{r_{\text{lu}}}+\frac{1}{r_{\text{ac}}+r_{\text{gs}}}}\right)V_{\text{d}}$
*E*_CUT_	ECOND-CUT = $\left(\frac{\frac{1}{r_{\text{lu}}}}{\frac{\left(1-W_{\text{st}}\right)}{\left(r_{\text{s}}+r_{\text{m}}\right)}+\frac{1}{r_{\text{lu}}}+\frac{1}{r_{\text{ac}}+r_{\text{gs}}}}\right)V_{\text{d}}$
*E*_SOIL_	ECOND-SOIL= $\left(\frac{\frac{1}{r_{\text{ac}}+r_{\text{gs}}}}{\frac{\left(1-W_{\text{st}}\right)}{\left(r_{\text{s}}+r_{\text{m}}\right)}+\frac{1}{r_{\text{ac}}+r_{\text{gs}}}+\frac{1}{r_{\text{ac}}+r_{\text{gs}}}}\right)V_{\text{d}}$
*E*_LCAN_	ECOND-LCAN = −9
*r*_b, stom_	RES-QLST = *r*_b_
*r*_b, cut_	RES-QLCT = *r*_b_
*r*_b, soil_	RES-QLSL= *r*_b_
*r*_b, lcan_	RES-QLLC= *r*_b_
*r*_dc_	RES-CONV = −9

**Table B6.** AQMEII4-reported gaseous deposition variables corresponding to the WRF-Chem resistance model of Fig. B6.

Name as described here	AQMEII4 name = resistance diagram variable name or formula
*r*_a_	Stable conditions : RES-AERO = $\frac{0.74\text{In}\left(\frac{z}{z_{0}}\right)+4.7\frac{z-z_{0}}{L}}{{\text{ku}}^{*}},\;z=2\;\text{m}.$
	Neutral conditions : RES-AERO = $\frac{0.74\ln\left(\frac{z}{z_{0}}\right)}{{\text{ku}}^{*}},\;z=2\;\text{m}$
	Unstable conditions: RES-AERO = $=\frac{0.74}{{\text{ku}}^{*}}\left\{\ln\;\left[\frac{\sqrt{1-9\frac{z}{L}}-1}{\sqrt{1-9\frac{z}{L}}+1}\right]-\ln\;\left[\frac{\sqrt{1-9\frac{z_{0}}{L}}-1}{\sqrt{1-9\frac{z_{0}}{L}}+1}\right]\;\right\}$
*r*_c_	RES-SURF = $\frac{1}{\frac{1}{r_{\text{m}}+r_{\text{S}}}+\frac{1}{r_{\text{cut}}}+\frac{1}{r_{\text{dc}}+r_{\text{cl}}}+\frac{1}{r_{\text{ac}}+r_{\text{gs}}}}$
*r*_s_	RES-STOM = $\text{ri}\;\left\{1+{\left(\frac{200}{\mathrm{Rad}+0.1}\right)}^{2}\right\}\frac{400}{T(40-T)}$
*r*_m_	RES-MESO = $\frac{1}{\frac{H}{3000}+100f_{\text{i}}}$
*r*_cut_	RES-CUT = *r*_lu_
*E*_STOM_	ECOND-ST = $\frac{1}{r_{\text{m}}+r_{\text{s}}}r_{\text{c}}V_{\text{d}}$
*E*_CUT_	ECOND-CUT = $\frac{1}{r_{\text{cut}}}r_{\text{c}}V_{\text{d}}$
*E*_SOIL_	ECOND-SOIL = $\frac{1}{r_{\text{ac}}+r_{\text{gs}}}r_{\text{c}}V_{\text{d}}$
*E*_LCAN_	ECOND-LCAN = $\frac{1}{r_{\text{dc}}+r_{\text{cl}}}r_{\text{c}}V_{\text{d}}$
*r*_b, stom_	RES-QLST = $2{\left({\text{ku}}^{*}\right)}^{-1}{\left(S_{c}/P_{r}\right)}^{2/3}$
*r*_b, cut_	RES-QLCT = $2{\left({\text{ku}}^{*}\right)}^{-1}{\left(S_{c}/P_{r}\right)}^{2/3}$
*r*_b, soil_	RES-QLSL = $2{\left({\text{ku}}^{*}\right)}^{-1}{\left(S_{C}/P_{r}\right)}^{2/3}$
*r*_b, lcan_	RES-QLLC = $2{\left({\text{ku}}^{*}\right)}^{-1}{\left(S_{c}/P_{r}\right)}^{2/3}$
*r*_dc_	RES-CONV = $100\left(1+\frac{1000}{\text{Rad}}\right)$

Prescribed values (table data) [pollutant, season]. *r*_cl_: for exposed surfaces in the lower canopy SO_2_, O_3_. *r*_ac_: for transfer that depends on canopy height and density. *r*_gs_: for ground surfaces SO_2_, O_3_. *r*_si_: for stomatal resistance. *r*_lu_: for outer surfaces in the upper canopy. *H*: Henry’s law constant. *f*_i_ : reactivity factor.

### B7 Example 7. COSMO-MUSCAT

**Table B7.** AQMEII4-reported gaseous deposition variables corresponding to the COSMO-MUSCAT resistance model of Fig. B8.

Name as described here	AQMEII4 name = resistance diagram variable name or formula
*r*_a_	RES_AERO = *r*_a_
*r*_c_	RES-SURF = ${\left({\left(r_{\text{S}}\right)}^{-1}+{\left(r_{\text{lu}}\right)}^{-1}+{\left(r_{\text{ac}}+r_{\text{gs}}\right)}^{-1}\right)}^{-1}$
*r*_s_	RES-STOM = *r*_s_
*r*_cut_	RES_CUT = *r*_lu_
*r*_gs_	RES_SOIL = *r*_gs_
*E*_STOM_	ECOND_ST = $\left(\frac{{\left(r_{\text{s}}\right)}^{-1}}{{\left(r_{\text{s}}\right)}^{-1}+{\left(r_{\text{lu}}\right)}^{-1}+{\left(r_{\text{in}}+r_{\text{gs}}\right)}^{-1}}\right)V_{\text{d}}$
*E*_CUT_	ECOND_CUT = $\left(\frac{{\left(r_{\text{lu}}\right)}^{-1}}{{\left(r_{\text{s}}\right)}^{-1}+{\left(r_{\text{lu}}\right)}^{-1}+{\left(r_{\text{in}}+r_{\text{gs}}\right)}^{-1}}\right)V_{\text{d}}$
*E*_SOIL_	ECOND_SOIL = $\left(\frac{{\left(r_{\text{in}}+r_{\text{gs}}\right)}^{-1}}{{\left(r_{\text{s}}\right)}^{-1}+{\left(r_{\text{lu}}\right)}^{-1}+{\left(r_{\text{in}}+r_{\text{gs}}\right)}^{-1}}\right)V_{\text{d}}$
*E*_LCAN_	ECOND-LCAN = −9
*r*_b, stom_	RES-QLST = *r*_b_
*r*_b, cut_	RES-QLCT = *r*_b_
*r*_b, soil_	RES-QLSL = *r*_b_

Resistances provided by COSMO (calculated by the “TKE-based surface transfer scheme” for water vapour). *r*_a_: aerodynamic resistance (depends on meteorology, not species). *r*_b_: quasi-laminar sublayer resistance (depends on gas diffusivity; H_2_O dependency is adjusted by gas diffusivity constants of the corresponding species). *r*_cut_: net cuticle resistance (“roughness layer” resistance over vegetation). *r*_ac_: resistance dependent only on canopy height and density (“roughness layer” resistance over non-vegetation).

### B7.1 The TKE-based surface transfer scheme (for water vapour)

The surface-layer scheme used in COSMO is intimately related to the TKE scheme. Here, the surface layer is defined to be the layer of air between the earth surface and the lowest model level. We subdivide the surface layer into a laminar-turbulent sub-layer, the roughness layer, and a constant-flux or Prandtl layer above. The roughness layer extends from the non-planar irregular surface, where the turbulent distance *l* = *λ/κ*(*λ*) is the turbulent length scale and *κ* is the von Kármán constant) is zero, up to a level *l* = *H*, such that *l* is proportional to the vertical height *z* within the Prandtl layer above. We choose to be equal to the dynamical roughness length *z*0. The lower boundary of the constant-flux layer (and of the atmospheric model) is defined to be a planar surface at a turbulent distance *l* = *H* from the surface. This subdivision allows us to discriminate between the values of the model variables at the rigid surfaces (predicted by the soil model) and values at the level *l* = *H*, which are “seen” by the atmosphere. For both layers, the fluxes are written in resistance form, where a roughness layer resistance is acting for scalar properties but not for momentum. Specific interpolation schemes are used to calculate the transport resistances of the layers. The applied surface scheme does not make use of empirical Monin–Obukhov stability functions, rather it generates these functions by the use of the dimensionless coefficients of the Mellor-Yamada closure and the interpolation rules.

The roughness layer is the region of the atmosphere into which vegetation and/or buildings protrude.

## Appendix C: Bidirectional ammonia fluxes

If a bidirectional flux algorithm for ammonia is employed in the model, then the flux may be either downwards (defined as positive here) or upwards (defined as negative, here). The generic equation for the bidirectional flux with this directionality is

(C1) $$F_{\text{T}}=\frac{c_{\text{a}}-c_{\text{c}}}{r_{\text{sum}}},$$

where *F*_T_ is the net flux, *c*_a_ and *c*_c_ are the atmospheric and canopy compensation point concentrations of ammonia gas, and *r*_sum_ is a sum of resistances. Different sources in the literature make use of different formulas for both *c*_c_ and *r*_sum_. For example, Zhang et al. (2010) employ

(C2) $$r_{\text{sum}}=r_{\text{a}}+r_{\text{b}},\;\text{and}\;c_{\text{c}}=\frac{\left(\frac{c_{\text{a}}}{r_{\text{a}}+r_{\text{b}}}+\frac{c_{\text{s}}}{r_{\text{s}}}+\frac{c_{\text{g}}}{r_{\mathrm{ac}}+r_{\mathrm{gs}}}\right)}{\left(\frac{1}{r_{\text{a}}+r_{\text{b}}}+\frac{1}{r_{\text{s}}}+\frac{1}{r_{\mathrm{ac}}+r_{\mathrm{gs}}}+\frac{1}{r_{\text{u}}}\right)},$$

where *c*_s_ and *c*_g_ are compensation point concentrations relative to stomata and ground, respectively, and all other terms are defined as above. In contrast, CMAQ with the M3dry deposition option uses (Bash et al., 2013; Pleim et al., 2013, 2019)

$$r_{\text{sum}}=r_{\text{a}}+0.5\;r_{\text{inc}},$$

where $r_{\text{inc}}=14\;\text{LAI}\frac{h_{\text{can}}}{u_{*}}$ (based on Erisman, 1994),

(C3) $$\text{and}\;c_{\text{c}}=\frac{-B+{\left(B^{2}-4AC\right)}^{0.5}}{2A},$$

where the variables A, B, and C in the quadratic of Eq. (C3) are given by

(C4) $$A=r_{\text{wet}}G_{t}\;B=r_{wb}G_{t}+\text{LAI}\left(1-f_{\text{wet}}\right)\;-r_{\text{wet}}\left(G_{\text{a}}c_{\text{a}}+G_{sb}c_{\text{s}}+G_{\text{g}}c_{\text{g}}\right)\;C=-r_{wb}\left(G_{\text{a}}c_{\text{a}}+G_{sb}c_{\text{s}}+G_{\text{g}}c_{\text{g}}\right).$$

The variables used to generate the coefficients in Eq. (C4) for the CMAQ M3dry option are given by

$$G_{\text{a}}=\frac{1}{r_{\text{a}}+0.5r_{\text{inc}}}\;G_{sb}=\frac{1}{r_{\text{s}}+r_{\text{b}}}\;G_{\text{g}}=\frac{1}{r_{bg}+0.5r_{\text{inc}}+r_{\text{soil}}}\;G_{t}=G_{sb}+G_{\text{g}}+G_{\text{a}}+f_{\text{wet}}G_{cw}\;G_{cw}=\frac{\text{LAI}}{r_{\text{b}}+r_{\text{wet}}}$$

(C5) $$r_{\text{wet}}=\frac{R_{\text{wo}}}{H_{\text{eff}}}\;r_{wb}=r_{\text{wet}}+\text{LAI}\;\left[a_{\text{h}}\left(1-f_{{\text{RH}}_{s}}\right)+r_{\text{b}}\right],$$

where the terms *r*_soil_, *H*_eff_, *a*_h_, *f*RH_s_, and *R*_wo_ are defined in Pleim et al. (2013). Note that in the latter reference (their Eq. 20), the summation term in Eq. (C4) above *G*_a_*c*_a_ is repeated twice within the bracketed terms (i.e. (*G*_a_c_a_ + *G*_*sb*_*c*_s_ + *G*_g_*c*_g_) as above is written (*G*_a_*c*_a_ + *G*_*sb*_*c*_s_ + *G*_a_*c*_a_ + *G*_g_*c*_g_), but this second occurrence of *G*_a_*c*_a_ is likely a typo).

CMAQ with the STAGE deposition option closely follows the widely used Massad et al. (2010) and Nemitz et al. (2001) parameterizations modified to include the option for a cuticular compensation point and employs the same resistance model for all deposited species as it reduced to RES-SURF from Table B3 when the stomatal, *C*_s_, cuticular, *C*_cut_, and ground, *C*_g_, compensation points are zero. NH_3_ bidirectional flux from the cuticle has been shown to be important (cuticular NH3 reference); however parameterizations applicable in a regional-scale model do not yet exist.

(C6) $$r_{\text{g}}=r_{\text{dc}}+r_{\text{gnd},\text{qlsb}}+r_{\text{gs}}$$

(C7) $$r_{\text{sum}}=r_{\text{a}}$$

(C8) $$c_{\text{c}}=\frac{\frac{c_{\text{a}}}{r_{\text{a}}}+\frac{c_{\text{leaf}}}{r_{\text{can},\text{qlsb}}}+\frac{c_{\text{g}}}{r_{\text{g}}}}{\frac{1}{r_{\text{a}}}+\frac{1}{r_{\text{can},\text{qlsb}}}+\frac{1}{r_{\text{dc}}+r_{\text{gnd},\text{qlsb}}+r_{\text{gs}}}}$$

*C*_leaf_ is the leaf compensation point and is estimated by solving for the exchange between the canopy compensation point and the atmosphere, stomata, cuticle, and ground following Kirchhoff’s current law (e.g. Nemitz et al., 2000). *C*_leaf_ is solved from this system of equations as follows.

(C9) $$c_{\text{leaf}}\frac{\frac{c_{\text{a}}}{r_{\text{a}}r_{\text{can},\text{qlsb}}}+\frac{c_{\text{s}}}{r_{\text{a}}r_{\text{s}}+r_{\text{can, qlsb}}r_{\text{s}}+r_{\text{g}}r_{\text{s}}}+\frac{c_{\text{cut}}}{r_{\text{a}}r_{\text{cut}}+r_{\text{can},\text{qlsb}}r_{\text{cut}}+r_{\text{g}}r_{\text{cut}}}+\frac{c_{\text{g}}}{r_{\text{dc}}+r_{\text{gnd},\text{qlsb}}+r_{\text{gs}}}}{\frac{1}{r_{\text{a}}r_{\text{can},\text{qlsb}}}+\frac{1}{r_{\text{a}}r_{\text{s}}}+\frac{1}{r_{\text{a}}r_{\text{cut}}}+\frac{1}{r_{\text{can},\text{qlsb}}r_{\text{s}}}+\frac{1}{r_{\text{can},\text{qlsb}}r_{\text{cut}}}+\frac{1}{r_{\text{can},\text{qlsb}}r_{\text{g}}}+\frac{1}{r_{\text{g}}r_{\text{s}}}+\frac{1}{r_{\text{g}}r_{\text{cut}}}}$$

The resistances *r*_cut_, *r*_can, qlsb_, and *r*_gnd, qlsb_ are taken from Massad et al. (2010). *r*_dc_ follows Shuttleworth and Wallace (1985) but integrating the canopy transport model of Yi (2008) using the in-canopy eddy diffusivity of Bash et al. (2010) from the soil surface to top of the canopy and assuming $r_{\text{a}}=p_{r}U/u_{*}^{2}$. The remainder of the resistances are the same as CMAQ with the M3dry deposition option.

(C10) $$r_{\text{dc}}=r_{\text{a}}\;\left(e^{\frac{\text{LAI}}{2}}-1\right)$$

Comparing approaches (Eqs. C2 through C10), *r*_sum_, *r*_a_, and *c*_c_ are held in common, and these approaches also make use of a stomatal (*c*_s_) and ground (*c*_g_) compensation point concentration, although how these terms are combined varies considerably between these approaches. For this reason, these common terms are reported as a separate TSD for ammonia bidirectional fluxes in AQMEII4 in order to allow cross-comparison of different approaches.

Note that the net flux of ammonia *F*_T_ appears as DFLUX-NH3 in the AQMEII4 documentation provided to participants as TSDs and may be positive or negative depending on direction. Ammonia values for *r*_b_, net canopy resistance, stomatal resistance, mesophyll resistance, cuticle resistance, and the three effective conductances also appear elsewhere in the TSDs, both for the grid scale and by AQMEII4 LULC category.
